# Systematic P2Y receptor survey identifies P2Y11 as modulator of immune responses and virus replication in macrophages

**DOI:** 10.15252/embj.2022113279

**Published:** 2023-10-26

**Authors:** Line Lykke Andersen, Yiqi Huang, Christian Urban, Lila Oubraham, Elena Winheim, Che Stafford, Dennis Nagl, Fionan O'Duill, Thomas Ebert, Thomas Engleitner, Søren Riis Paludan, Anne Krug, Roland Rad, Veit Hornung, Andreas Pichlmair

**Affiliations:** ^1^ Institute of Virology, School of Medicine Technical University of Munich Munich Germany; ^2^ Institute of Immunology, Biomedical Center Ludwig‐Maximilians‐Universität München Munich Germany; ^3^ Department of Biochemistry, Gene Center Munich Ludwig‐Maximilians‐Universität München Munich Germany; ^4^ Institute of Molecular Oncology and Functional Genomics, School of Medicine Technical University of Munich Munich Germany; ^5^ Department of Biomedicine Aarhus University Aarhus Denmark; ^6^ Center of immunology of viral infection (CiViA) Aarhus University Aarhus Denmark; ^7^ German Center for Infection Research (DZIF), Munich Partner Site Munich Germany

**Keywords:** antiviral immunity, cytokine induction, innate immunity, nucleotide sensing, P2YR, Immunology, Microbiology, Virology & Host Pathogen Interaction

## Abstract

The immune system is in place to assist in ensuring tissue homeostasis, which can be easily perturbed by invading pathogens or nonpathogenic stressors causing tissue damage. Extracellular nucleotides are well known to contribute to innate immune signaling specificity and strength, but how their signaling is relayed downstream of cell surface receptors and how this translates into antiviral immunity is only partially understood. Here, we systematically investigated the responses of human macrophages to extracellular nucleotides, focusing on the nucleotide‐sensing GPRC receptors of the P2Y family. Time‐resolved transcriptomic analysis showed that adenine‐ and uridine‐based nucleotides induce a specific, immediate, and transient cytokine response through the MAPK signaling pathway that regulates transcriptional activation by AP‐1. Using receptor trans‐complementation, we identified a subset of P2Ys (P2Y1, P2Y2, P2Y6, and P2Y11) that govern inflammatory responses via cytokine induction, while others (P2Y4, P2Y11, P2Y12, P2Y13, and P2Y14) directly induce antiviral responses. Notably, P2Y11 combined both activities, and depletion or inhibition of this receptor in macrophages impaired both inflammatory and antiviral responses. Collectively, these results highlight the underappreciated functions of P2Y receptors in innate immune processes.

## Graphical abstract

Most human nucleotide‐sensing P2Y macrophage receptors are involved either in cytokine induction or antiviral responses, while P2Y11 uniquely combines these two roles.

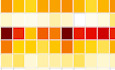

## Introduction

The immune system consists of inter‐ and intracellular signaling networks that sense both pathogen‐associated molecular patterns (PAMPs) and damage‐associated molecular patterns (DAMPs) through pattern recognition receptors (PRRs). While PAMPs are molecules delivered by pathogens during infection, DAMPs are host‐derived molecules that are exposed during cellular stress or damage. Prototypical DAMPs include proteins such as high‐mobility group box 1 (HMGB1), S100 proteins, and heat shock proteins (HSPs; Matzinger, 1994; Medzhitov, 2007; Gong *et al*, 2020). Notably, extracellular nucleotides released from damaged or dying cells can also be recognized as DAMPs by cell surface purinergic receptors (P2Rs). The P2Rs are divided into ionotropic P2X receptors (P2XRs) and metabotropic P2Y receptors (P2YRs; Burnstock & Kennedy, 1985). P2XRs are classical ATP‐gated channels composed of seven major members (P2X_1–7_), which form homo‐ or heterotrimeric ion channels that are permeable for Na^+^, K^+^, and Ca^2+^ ions (Khakh *et al*, 2001). The P2YR family consists of eight G protein‐coupled receptors (GPCRs) P2Y_1_, P2Y_2_, P2Y_4_, P2Y_6_, P2Y_11_, P2Y_12_, P2Y_13_, and P2Y_14_ and are, as opposed to the P2XRs, activated by a wide range of nucleotides. P2Y_1_, P2Y_12_, and P2Y_13_ are activated by ADP, while the ligand for P2Y_11_ is ATP. UTP, UDP, and UDP‐glucose serve as ligands for P2Y_4_, P2Y_6_, and P2Y_14_, respectively. In contrast, P2Y_2_ is equally sensitive to both ATP and UTP (Jacobson *et al*, 2020).

P2R signaling regulates multiple aspects of immune cell‐mediated inflammation. For instance, sensing of ATP by P2X_7_ was shown to be involved in release of several cytokines and chemokines, where the most described effect is as a driver of inflammasome‐mediated secretion of IL‐1β in mouse macrophages (Ferrari *et al*, 1997; Solle *et al*, 2001; Qu *et al*, 2007; Di Virgilio *et al*, 2017). It is also established that P2Y_2_ and P2Y_6_ are involved in the secretion of chemokines from macrophages, in particular by enhancing PAMP‐induced chemokine release (Kukulski *et al*, 2007; Stokes & Surprenant, 2007; Ben Yebdri *et al*, 2009; Kim *et al*, 2011). Furthermore, P2Y_2_ and P2Y_6_ modulate chemotaxis of neutrophils and monocytes toward these chemokines and other chemoattractants through autocrine signaling (Kukulski *et al*, 2009; Kronlage *et al*, 2010; Campwala *et al*, 2014; Bao *et al*, 2015). Moreover, autocrine signaling through P2Y_11_ supports T‐cell chemotaxis and activation (Ledderose *et al*, 2020, 2021). P2R‐dependent immune cell recruitment and activation play an important role in the pathogenesis of multiple inflammatory diseases such as asthma, chronic obstructive pulmonary disease, atherosclerosis, multiple sclerosis, inflammatory bowel disease, and rheumatoid arthritis (Antonioli *et al*, 2019). In the context of viral infections, P2Rs appear to be functionally heterogeneous, suggested by reports of both pro‐ and antiviral activities (Eberhardt *et al*, 2022). For instance, P2X_7_ activation by ATP released in response to viral infection positively regulates the expression and release of IFN‐β, which is crucial in promoting immunity against several viruses (Tsai *et al*, 2015; Zhang *et al*, 2017). P2X_7_ signaling also contributes to excessive inflammation during influenza and adenovirus infection, thereby leading to higher mortality (Lee *et al*, 2012; Leyva‐Grado *et al*, 2017). P2Y_2_ promotes the replication of human cytomegalovirus (HCMV; Chen *et al*, 2019) and human immunodeficiency virus type 1 (HIV‐1; Seror *et al*, 2011; Paoletti *et al*, 2019), whereas it ameliorates pneumonia virus of mice (PVM) infection in mice (Vanderstocken *et al*, 2012). P2Y_6_ was linked to protection of mice from vesicular stomatitis virus (VSV) infection (Li *et al*, 2014), while P2Y_13_ restricts replication of VSV, Newcastle disease virus (NDV), and Herpes simplex virus 1 (HSV‐1; Zhang *et al*, 2019).

The previous studies reporting the P2YR involvement in the immunopathology of multiple diseases and diverse pro‐ and antiviral roles were mostly studies of individual P2YRs in specific responses. Although varying in combination depending on the cell type, several P2YRs are expressed on the same cell and collectively contribute to regulating cell type‐specific responses. Thus, we performed a comprehensive comparative analysis of immune‐regulatory functions of P2YRs with respect to their ligands. We systematically studied their expression in different cell types and tested for their involvement in cytokine release and viral restriction. Notably, we found that P2YRs can be classified into two sets of receptors: receptors that regulate cytokine expression in an AP‐1‐dependent manner and receptors that regulate virus growth. From this comparative dataset, we identified P2Y_11_ as the receptor predominantly important for macrophages to regulate cytokine expression and virus replication in response to nucleotide engagement.

## Results

### Expression patterns of P2YRs on commonly used cell types

**Figure 1:**
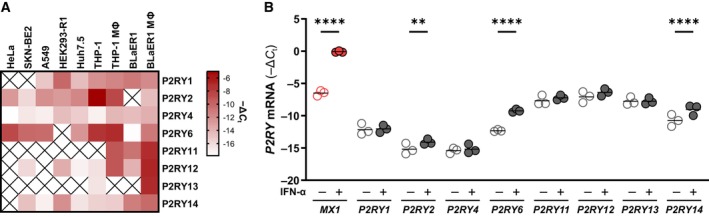
P2RY expression levels in various cell lines A, BTotal RNA from HeLa, SKN‐BE2, A549, HEK293‐R1, Huh7.5, THP‐1, and BLaER1 cells and differentiated THP‐1 and BLaER1 cells (A) or differentiated BLaER1 cells untreated or treated with 250 U/ml IFN‐α for 8 h (B) were analyzed for the endogenous *P2RY* levels by RT–qPCR. The *P2RY* levels were normalized to GAPDH and the mean of three independent experiments shown as a heat map (A) or as individual values together with the mean and ****P < 0.0001, ***P < 0.001, **P < 0.01 (two‐way ANOVA with Šídák's multiple comparison test) (B). Source data are available online for this figure.

To investigate the immune‐regulatory effects of P2Y activation by nucleotides, we first evaluated P2YR mRNA expression in various nonimmune (HeLa, HEK293‐R1, A549, SKN‐BE2, and Huh7.5) and immune (THP‐1 monocytes, THP‐1‐derived macrophages, BLaER1 B cells, and BLaER1‐derived macrophages) cell lines (Fig 1A). P2YR mRNA expression levels revealed that *P2RY1*, *P2RY2*, *P2RY4*, *P2YR6*, *P2RY12*, and *P2RY14* were expressed in the majority of cells. However, with the exception of *P2RY6*, the P2RY expression levels were very low in most of the nonimmune cell lines. *P2RY2* was highly expressed specifically in THP‐1 monocytes and THP‐1‐derived macrophages. The macrophages derived from THP‐1 and BLaER1 cells expressed considerable levels of additional P2YRs, namely *P2RY11* and *P2RY12*, whereas high levels of *P2RY13* were exclusively detected in BLaER1‐derived macrophages (Fig 1A). From these experiments, we concluded that the expression patterns of P2YRs are diverse, and we selected THP‐1‐ and BLaER1‐derived macrophages for further studies since they expressed the broadest repertoire of P2YRs.

The expression of many proteins with antiviral functions is induced by interferon (IFN)‐α/β signaling, and notably, P2Y_6_ was previously described to be induced by IFN‐α (Hubel *et al*, 2019). To characterize the IFN inducibility of all P2YRs, we stimulated BLaER1‐ and THP‐1‐derived macrophages with IFN‐α and quantified P2YR mRNA expression (Fig 1B and Appendix Fig S1). As expected, IFN‐α treatment led to the induction of the interferon‐stimulated genes (ISGs) *MX1* and *P2RY6*. Other than *P2RY6*, most P2YRs did not show elevated expression after IFN‐α treatment. A notable exception was *P2RY14*, which we identified as an ISG in both THP‐1‐ and BLaER1‐derived macrophages (Fig 1B and Appendix Fig S1). To our knowledge, P2Y_14_ has not been reported to be an ISG, likely due to the lack of, or very low, expression in most commonly used cell lines.

### P2YR ligands induce a cytokine response in macrophages

To investigate the potential role of P2YRs in an unbiased manner, we stimulated differentiated BLaER1 cells with a selection of nucleotides (diadenosine tetraphosphate (Ap_4_A), ATP, ADP, UTP, and UDP), which were previously reported to activate P2YRs (Nicholas *et al*, 1996; Palmer *et al*, 1998; Communi *et al*, 1999, 2001; Chambers *et al*, 2000; Hollopeter *et al*, 2001; Jacobson *et al*, 2020). The differentiation of the BLaER1 cells into macrophages was confirmed by the upregulated mRNA expression of five different macrophage markers compared with the undifferentiated cells (Appendix Fig S2A). Since human macrophages are highly sensitive to PAMP stimulation, we tested the synthetic nucleotides for potential impurities by endotoxin that would result in the activation of TLR4‐dependent NF‐κB signaling. Unexpectedly, many commercially available nucleotides activated a TLR4‐NF‐κB reporter cell line and we proceeded all further experiments using nucleotides that were not activating TLR4 (Appendix Fig S2B). Moreover, we ensured that the tested nucleotides did not affect cell viability. Of all the nucleotides, only ADP reduced the cell viability signal, which is likely due to substrate‐dependent inhibition of the CellTiter‐Glo system (Appendix Fig S2C).

**Figure 2:**
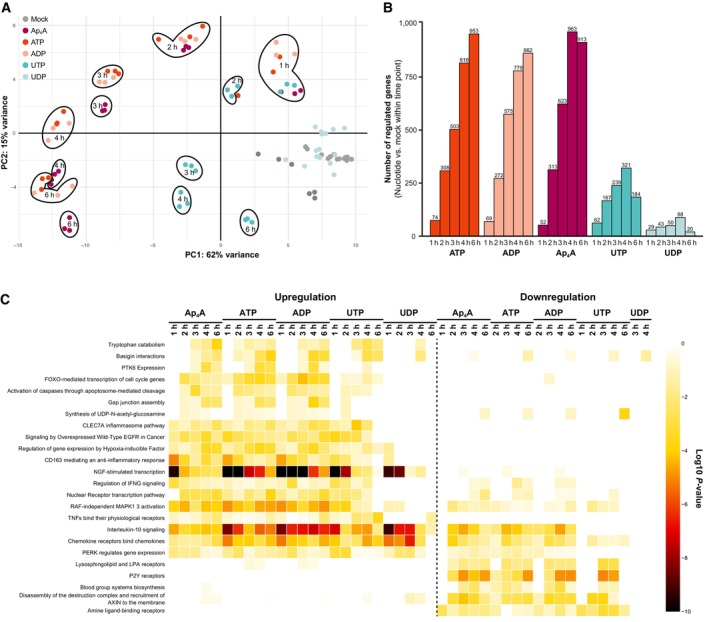
Nucleotides induce changes to the transcriptome over time Transcriptomic analysis of differentiated BLaER1 cells treated with 500 μM Ap_4_A, ATP, ADP, UTP, or UDP for 1, 2, 3, 4, or 6 h.
Principal component analysis (PCA) on global gene expression profiles of individual biological replicates. Colors represent different treatment groups, and axes represent the first two components.The number of distinct transcripts that are significantly regulated (adjusted *P*‐value ≤ 0.01, log2 fold change ≥ 1) at given time points after treatment relative to untreated cells at the same time point.Gene set enrichment analysis of the regulated transcripts from (B) using Reactome database (Fisher's exact test unadjusted *P*‐value ≤ 0.001).

To decipher the cellular transcriptional response to nucleotide signaling, we stimulated BLaER1 macrophages with Ap_4_A, ATP, ADP, UTP, and UDP or left them untreated and performed a time‐resolved transcriptome analysis (Fig 2A–C, and Datasets EV1 and EV2). We quantified more than 20,000 genes, of which 2,110 were found to be significantly regulated by treatment with one or more nucleotides relative to time‐matched untreated conditions. Principle component analysis (PCA) verified high similarity between replicates and furthermore revealed the existence of distinct transcriptional signatures induced by stimulation with specific nucleotides (Fig 2A). As expected, untreated cells did not display strong deviations at any measured time point. Similarly, UDP‐stimulated samples clustered with untreated samples, indicating that UDP induced only minor transcriptional changes. In contrast, Ap_4_A, ATP, ADP, and UTP‐stimulated samples clearly segregated from mock samples across the assessed time frame. Notably, transcriptional changes induced by adenine‐derived nucleotides Ap_4_A, ATP, and ADP were highly similar to each other and followed comparable trajectories over time. However, although initially following the same trend, Ap_4_A starts to segregate from the ATP/ADP cluster after 3 h. This could be due to the structural specificities of Ap_4_A leading to a more pronounced regulation of the transcripts particularly at later time points (Fig 2A). For all nucleotides used, the number of regulated genes compared with mock increased over time. In line with the changes observed in the PCA, cells stimulated with adenine‐derived nucleotides ATP, ADP, and Ap_4_A displayed higher numbers of significantly regulated genes as compared to the uridine‐derived nucleotides UTP and UDP (Fig 2B).

**Figure 3:**
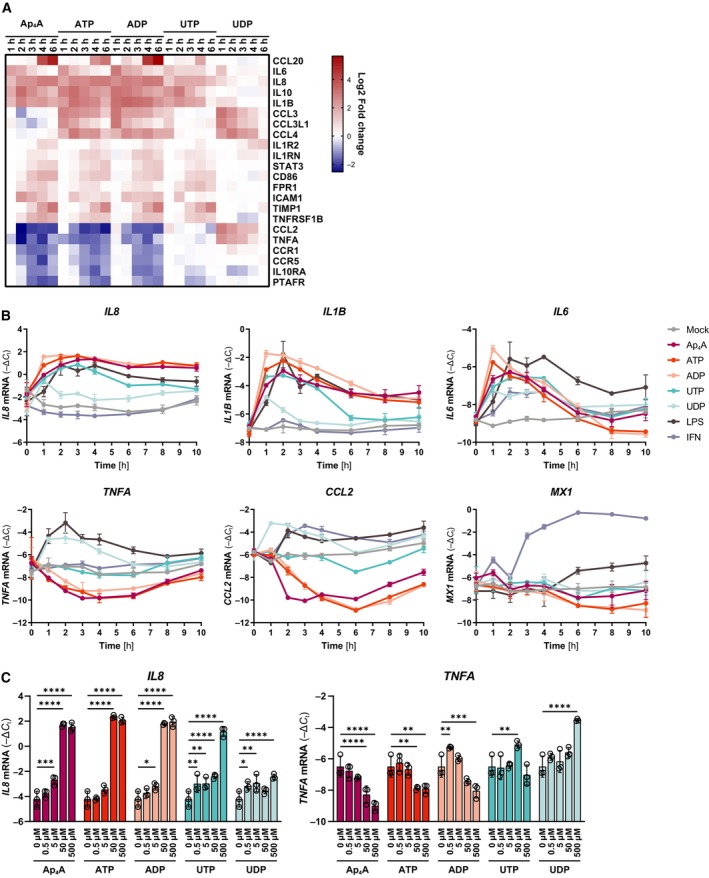
Adenine‐derived nucleotides induce interleukin gene expression AHeat map of the log2 fold change gene expression (treatment vs. mock within a time point) for transcripts within the interleukin signaling Reactome term from Fig 2C.B, CDifferentiated BLaER1 cells treated with 500 μM Ap_4_A, ATP, ADP, UTP, or UDP, 0.1 ng/ml LPS or 200 U/ml IFN‐α for the indicated time points (B) or various concentrations of Ap_4_A, ATP, ADP, UTP, or UDP for 2 h (C) and the levels of the indicated cytokines analyzed by RT–qPCR. The cytokine levels were normalized to *GAPDH* and the mean ± SD of three biological replicates shown. The data presented are a representative of two independent experiments. ****P < 0.0001, ***P < 0.001, **P < 0.01, *P < 0.05 (two‐way ANOVA with Dunnett's multiple comparison test). Source data are available online for this figure.

We investigated the features of the differentially expressed genes by Reactome pathway enrichment analysis to identify biological pathways and responses regulated by stimulation with distinct nucleotides (Gillespie *et al*, 2022). Overall, we found that most of the strongly enriched pathways were related to immunity, for example, TNF, chemokine, and interleukin signaling with the latter being particularly prominently regulated (Fig 2C). Interestingly, stimulation with Ap_4_A, ATP, ADP, and UTP led to a rapid induction of a subset of pro‐inflammatory cytokines such as *IL8* (*CXCL8*), *IL1B*, *and IL6* already at 1 h after stimulation, while expression of this set of cytokines was not affected by UDP stimulation (Fig 3A). Despite relative low number of transcriptional changes induced by UDP treatment, UDP caused upregulation of *TNFA* and *CCL2*, which were downregulated by Ap_4_A, ATP and ADP and not changed by UTP. The PCA showed that stimulation with Ap_4_A separated from treatment with ATP and ADP. This separation was evident from another group of cytokines containing *CCL3* and *CCL4*, which were regulated by ATP, ADP, and UDP, but not Ap_4_A or UTP (Fig 3A). Notably, we could not identify differential regulation of ISGs (Fig 2C), which is a hallmark of antiviral responses (Schoggins, 2019).

We systematically validated the nucleotide‐induced upregulation of a set of cytokines by RT–qPCR, also using the TLR4 ligand LPS and recombinant IFN‐α as references (Fig 3B). In line with the transcriptomics data, Ap_4_A, ATP, ADP, and UTP led to rapid upregulation of *IL8*, *IL1B*, and *IL6*, peaking at 1‐ to 2 h poststimulation. In accordance with the transcriptome data, expression of *IL8* mRNA was upregulated 1 h after stimulation with the aforementioned nucleotides and stayed high until 10 h poststimulation. In contrast, the extended kinetic analysis revealed that the upregulation of *IL6* and *IL1B* was of transient nature and that their mRNA abundances gradually declined after an initial burst of expression 1 h poststimulation. The prototypical PAMP LPS similarly upregulated all three cytokines, but with a slightly slower kinetics. A prominent difference between nucleotide and LPS stimulation was evident for *TNFA* and *CCL2*. While the adenine‐derived nucleotides downregulated *TNFA* and *CCL2*, LPS upregulated both transcripts. Moreover, in contrast to LPS stimulation, nucleotides did not induce an IFN response over time, as judged from the expression of the ISG *MX1*. The presence of a functional IFN response in the BLaER1 macrophages was further validated by the strong upregulation of *MX1* and *CCL2* upon IFN‐α treatment (Fig 3B; Lehmann *et al*, 2016). In line with the transcriptomics analysis (Fig 3A), UDP markedly upregulated *TNFA* and *CCL2*, while induction of *IL8*, *IL1B*, and *IL6* was not as prominent (Fig 3B). We further validated the integrity of transcriptional upregulation of *IL8* in differentiated *TLR4* knockout BLaER1 cells (Appendix Fig S3A). The nucleotide‐dependent *IL8* response was also apparent in *TLR4* knockout cells, suggesting that the response was not triggered by any residual endotoxin contamination (Appendix Fig S3A). Moreover, ATP has been reported to induce cytokines through P2X_7_. However, the P2X_7_‐specific antagonist A438079 (Donnelly‐Roberts & Jarvis, 2007) did not affect either the upregulation of *IL8* or the downregulation of *TNFA* in response to treatment of differentiated BLaER1 cells with Ap_4_A, ATP, or ADP, ruling out contribution of P2X_7_ activation to the adenine‐derived nucleotide‐driven transcriptional responses (Appendix Fig S3B).

In the transcriptomics analysis as well as RT–qPCR validations, we used nucleotides at a concentration of 500 μM. To test the sensitivity of the observed responses, we treated differentiated BLaER1 cells with graded doses of nucleotides for 2 h and quantified *IL8* and *TNFA* mRNA abundances (Fig 3C) alongside cell viability (Appendix Fig S2C). Significant differences in the cellular responses to adenine‐derived nucleotides required at least 5 μM and saturated at 50 μM, while UTP required 10‐fold higher concentrations to produce a response in this assay. Similarly, the induction of *TNFA* by UDP was only apparent at the highest concentration (Fig 3C).

**Figure 4:**
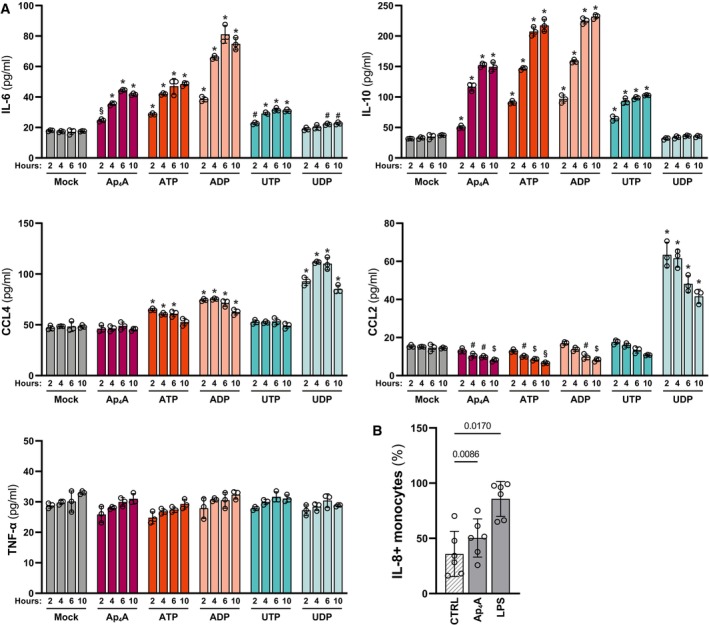
Adenine‐derived nucleotides induce interleukin release Differentiated BLaER1 cells treated with 500 μM Ap4A, ATP, ADP, UTP, or UDP for 2, 4, 6, or 10 h and analyzed for cytokine release by cytometric bead array. The graph shows the cytokine levels from three biological replicates together with mean ± SD. *P < 0.0001, ^§^ *P* < 0.001, ^$^ *P* < 0.01, ^#^ *P* < 0.05 (two‐way ANOVA with Dunnett's multiple comparison test, comparison to mock).PBMCs isolated from six donors treated with 500 μM Ap_4_A or 10 ng/ml LPS for 8 h with protein secretion blocked after the initial 4 h. The cells were stained for viability using a fluorescent cell permeability dye and intracellular IL‐8 and surface CD14 (monocytes) using specific fluorescently coupled antibodies and analyzed by flow cytometry. The percentage of IL‐8^+^ CD14^+^ cells of the total CD14^+^ cells is shown for each donor together with the mean ± SD and *P*‐value (one‐way repeated measures ANOVA with Dunnett's multiple comparison test).

We next investigated whether nucleotide stimulation of differentiated BLaER1 cells leads to cytokine secretion. Differentiated BLaER1 cells were treated with Ap_4_A, ATP, ADP, UTP, or UDP for 2, 4, 6, and 10 h and a panel of cytokines detected in the supernatant using cytometric bead array (CBA). In accordance with the transcriptomic regulation (Fig 3A and B), IL‐6 and IL‐10 were secreted in a time‐dependent manner in response to Ap_4_A, ATP, ADP, and UTP, but not UDP (Fig 4A). The baseline secretion of IL‐8 in the BLaER1 macrophages was too high and exceeded the maximum range of the CBA (Dataset EV3). CCL4 was specifically transcribed in ATP‐, ADP‐, and UDP‐stimulated cells, and this selective induction could also be reflected in CCL4 secretion levels. The downregulation of CCL2 by adenine‐derived nucleotides and upregulation by UDP could also be observed (Fig 4A). We could not observe differential secretion of IL‐1β, IL‐2, IL‐5, IL‐7, IL‐12 (p70), G‐CSF, GM‐CSF, TNF‐α, or IFN‐γ after stimulation with any of the nucleotide tested (Dataset EV3).

To further corroborate these findings in primary cells *ex vivo*, we stimulated PBMCs from six different donors with Ap_4_A and quantified intracellular IL‐8 abundance in the different immune cell subsets (Fig 4B and Appendix Fig S3C) using flow cytometry. Ap_4_A treatment significantly upregulated IL‐8 in CD14^+^ monocytes as compared to treatment with vehicle (Fig 4B). Notably, there was no induction of intracellular IL‐8 in CD4^+^ T cells, CD8^+^ T cells, B cells, natural killer (NK) cells, conventional dendritic cells (cDCs), or plasmacytoid DCs (pDCs; Appendix Fig S3C). This indicates that the responses to nucleotides observed in BLaER1 macrophages are functional in *ex vivo* macrophages and that this response is cell type restricted.

Collectively, these data revealed an underappreciated diversity of cytokine expression patterns, whereby adenine‐derived nucleotides behave similarly, but are clearly distinct from uridine‐derived nucleotides. Furthermore, the tested nucleotides feed into signaling pathways that are different from the ones induced by the prototypical PAMP LPS and thus produce distinct inflammatory responses.

### Nucleotides induce interleukins independent of the NF‐κB pathway

**Figure 5:**
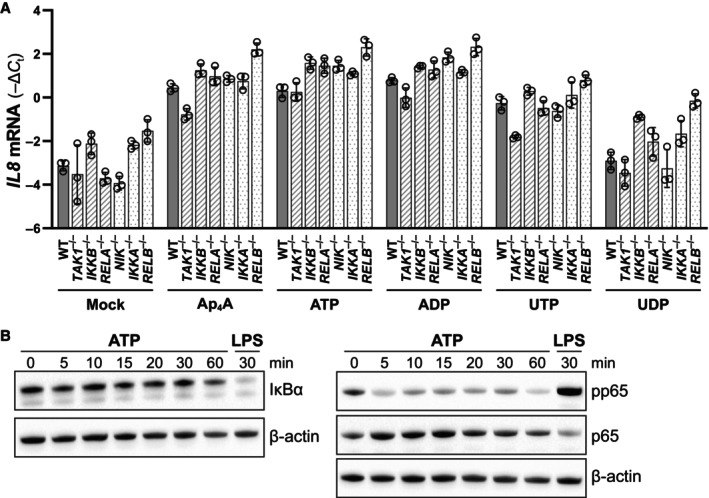
P2YRs do not activate NF‐κB signaling Differentiated BLaER1 WT and *TAK1*, *IKKB*, *RELA*, *NIK*, *IKKA*, or *RELB* KO cells treated with 500 μM Ap_4_A, ATP, ADP, UTP, or UDP for 2 h and analyzed for *IL8* levels using RT–qPCR. The *IL8* levels were normalized to *GAPDH* and the mean ± SD of three biological replicates shown. The data presented are a representative of two independent experiments.Differentiated BLaER1 cells treated with 500 μM ATP for the indicated times followed by western blotting against IκBα, phosphorylated and total p65 (RELA) and β‐actin. Source data are available online for this figure.

We next investigated signaling cascades that may be activated by nucleotide treatments. Expression of some of the nucleotide‐induced interleukins, for example, *IL6*, *IL8*, and *IL10*, is known to be regulated by the transcription factor NF‐κB. NF‐κB can be activated through either the canonical or the noncanonical pathway. In the canonical pathway, TAK1 activates the complex of IKK‐α, IKK‐β and IKK‐γ, which leads to degradation of IκBα, releasing the NF‐κB p50/p65 (RELA) dimer. The noncanonical pathway relies on NIK that activates IKK‐α, which phosphorylates and activates the p52/RELB NF‐κB dimer (Mitchell *et al*, 2016). To test for the involvement of the NF‐κB pathways in nucleotide‐induced cytokine responses, the central NF‐κB signaling components TAK1 (MAP3K7), IKK‐β (IKBKB), RELA, NIK (MAP3K14), IKK‐α (CHUK), and RELB were depleted in BLaER1 cells. We then stimulated differentiated BLaER1 knockouts with the various nucleotides for 2 h. The overall viability of the differentiated knockout cells was not affected (Appendix Fig S4A). Surprisingly, genetic depletion of the central components of neither canonical (TAK1, IKK‐β, nor RELA) nor noncanonical (NIK, IKK‐α, or RELB) NF‐κB signaling affected *IL8* induction in response to the nucleotides (Fig 5A). Moreover, while LPS stimulation led to hallmark NF‐κB activation as illustrated by phosphorylation of p65 (RELA) and degradation of IκBα, stimulation with ATP, despite similar response kinetics (Fig 3C), did not (Fig 5B). Altogether, this shows that the transcription of the cytokines in response to nucleotide stimulation must be driven by an activation mechanism other than NF‐κB signaling.

### Nucleotides induce interleukins through P2YR‐mediated MAPK activation of AP‐1

**Figure 6:**
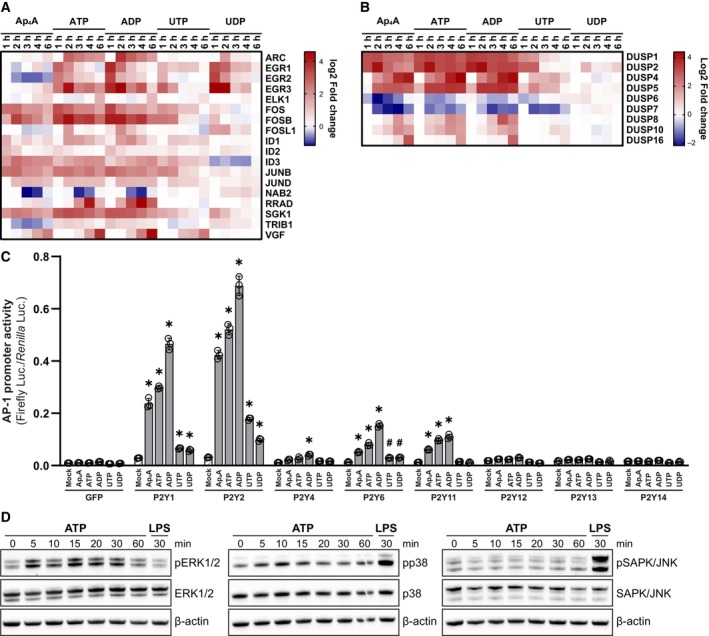
P2YRs activate MAPK‐AP‐1 signaling A, BHeat maps of the log2 fold change gene expression (treatment vs. mock within a time point) for transcripts within the NGF transcription factor (A) and RAF‐independent MAPK1‐3 signaling (B) Reactome terms from Fig 2C.CHEK293‐R1 cells transfected with individual P2YRs together with AP‐1 promoter Firefly luciferase and EF‐1α promoter *Renilla* reporter plasmids and then treated with 1 μg/ml doxycycline for 8 h followed by 500 μM Ap_4_A, ATP, ADP, UTP, or UDP for 16 h. The graph shows the Firefly/*Renilla* signal from three biological replicates together with mean ± SD. *P < 0.0001, ^§^ *P* < 0.001, ^$^ *P* < 0.01, ^#^ *P* < 0.05 (two‐way ANOVA with Dunnett's multiple comparison test, comparison to mock). The data presented are a representative of three independent experiments.DDifferentiated BLaER1 cells treated with 500 μM ATP for the indicated times followed by western blotting against phosphorylated and total ERK1/2, p38 and SAPK/JNK and β‐actin. Source data are available online for this figure.

To identify potential signaling mechanisms activated by the nucleotides, we revisited the transcriptomics data, in particular the Reactome pathways that were enriched upon nucleotide treatments (Fig 2C). The most significantly upregulated term NGF‐stimulated transcription contained genes for multiple transcription factors, among them were several members of the activator protein‐1 (AP‐1) transcription factor family such as FOS, FOSB, JUNB, and JUND (Fig 6A). AP‐1 transcription factors are known to positively affect their own transcription, suggesting their involvement in the nucleotide‐induced transcriptional responses (Karin, 1995). Notably, AP‐1 transcription factor expression was strongly upregulated by treatment with Ap_4_A, ATP, ADP, and UTP (Fig 6A) with similar kinetics to the interleukins *IL8*, *IL6*, *IL1B*, and *IL10* (Fig 3A). Additionally, we could identify rapid and intense regulation of dual‐specificity MAPK phosphatases (DUSPs) within the term RAF‐independent MAPK1‐3 activation (Figs 2C and 6B). DUSPs are negative regulators of MAPKs, which among others serve as kinases upstream of AP‐1 activation (Karin, 1995; Dickinson & Keyse, 2006). More specifically, we identified early downregulation of the cytoplasmic DUSP6 and −7 in response to Ap_4_A, ATP, ADP, and UTP. Moreover, the same nucleotides induced expression of the nuclear DUSP1, −2, −4, and −5, and with some delay DUSP8, −10, and −16 that can be both cytoplasmic and nuclear (Fig 6B). We thereby hypothesized that the nucleotide signaling may initially activate MAPK‐dependent AP‐1 signaling leading to elevated cytokine expression, which is then terminated through a delayed negative feedback loop whereby DUSPs dephosphorylate the MAPKs in the nucleus.

To investigate whether the P2YRs can induce AP‐1 activation in response to nucleotides, we opted to trans‐complement HEK293‐R1 cells with the P2YRs. Briefly, we transfected HEK293‐R1 cells with doxycycline‐inducible expression plasmids encoding different P2YRs together with a reporter plasmid for Firefly luciferase driven by the AP‐1 promoter. We first tested for expression of the individual P2YRs by western blot (Appendix Fig S4B). Upon doxycycline induction, all P2YRs were expressed, although P2Y_1_, P2Y_2_, and P2Y_4_ were more abundant than P2Y_6_, P2Y_11_, P2Y_12_, P2Y_13_, and P2Y_14_ (Appendix Fig S4B). The transfected and doxycycline‐treated cells were then stimulated with Ap_4_A, ATP, ADP, UTP, UDP, or with vehicle, and luciferase levels were measured 16 h later (Fig 5C). Strikingly, this experiment revealed a remarkable diversity of P2YRs' ability to activate AP‐1. While cells transfected with control plasmid (GFP) did not respond to treatment with nucleotides, P2Y_1_, P2Y_2_, P2Y_6_, and P2Y_11_ expressing cells strongly activated the AP‐1 promoter in a nucleotide treatment‐dependent manner (Fig 6C), with the absolute levels correlating with the expression levels of the respective transcomplemented receptors (Appendix Fig S4B). In contrast, we could not detect any substantial AP‐1 activation in cells that expressed P2Y_4_, P2Y_12_, P2Y_13_, or P2Y_14_ (Fig 6C). The P2YR‐dependent activation was most prominent in response to adenine‐derived nucleotides except for P2Y_2_‐expressing cells, which also modestly responded to UTP and UDP. Among the nucleotides tested, ADP was the most potent inducer, followed by ATP and Ap_4_A. (Fig 6C). The preference of adenine‐derived nucleotides in inducing P2Y‐dependent AP‐1 promoter activation correlated with the ability of those nucleotides to induce transcription of cytokines and AP‐1 transcription factors in the transcriptome analysis (Figs 3A and 6A).

Since AP‐1 transcription factors are activated by MAPKs, we tested the activation of the classical MAPKs ERK1/2, p38, and JNK by phosphorylation‐specific western blot analysis of BLaER1 macrophages treated with ATP for 5 to 30 min (Fig 6D). We detected an increased abundance of phosphorylated ERK1/2 and p38 already after 5 min of ATP stimulation. The phosphorylation of ERK was sustained for 30 min and returned to baseline within 60 min after stimulation. In contrast, p38 activation was only transient and terminated within 15 min. We could not observe phosphorylation of JNK in the timespan tested. In contrast, LPS stimulation led to phosphorylation of p38 and JNK, indicating induction of a stronger and longer lasting signal by this treatment (Fig 5D).

Collectively, the transcriptomics data in conjunction with the findings on profiling AP‐1 and MAPK activation indicated that the used nucleotides stimulate interleukin production through MAPK‐induced AP‐1 transcriptional activation. P2Y_1_, P2Y_2_, P2Y_6_, and P2Y_11_ have the capability of activating the AP‐1 promoter and could thus be the receptors that shape immunomodulatory activities in response to extracellular nucleotides.

### P2Y_11_ is involved in the macrophage nucleotide response

**Figure 7:**
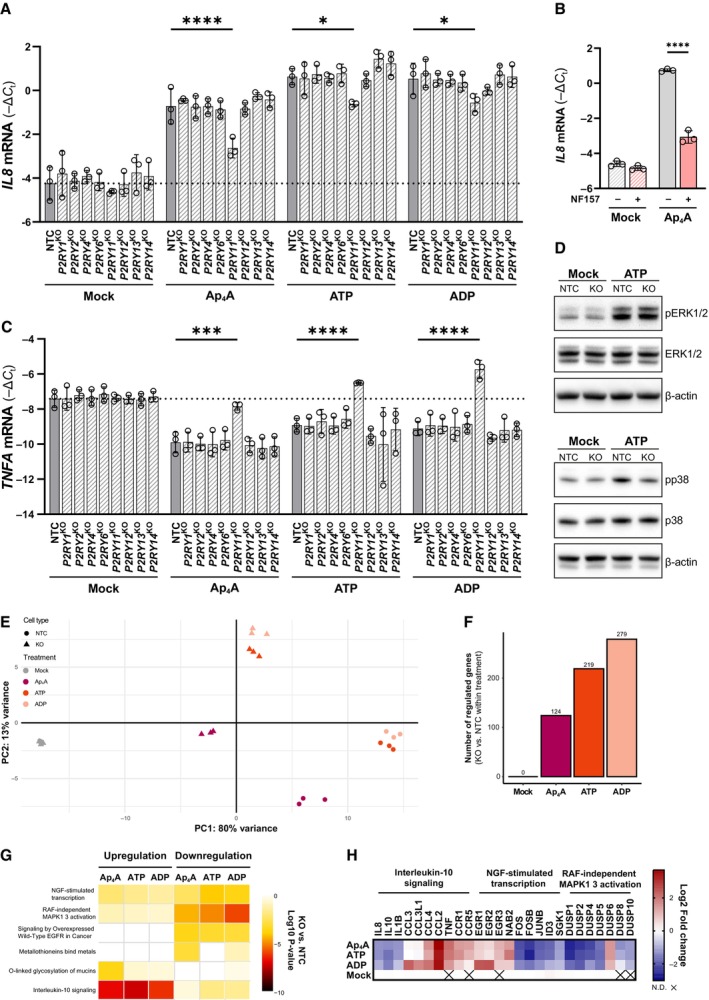
P2Y_11_ is responsible for the cytokine expression profile in macrophages A–CDifferentiated BLaER1 nontargeted control (NTC) and *P2RY* KO cells treated with 500 μM Ap_4_A, ATP, or ADP for 2 h (A + C) or differentiated BLaER1 cell pretreated with 100 μM P2Y_11_ antagonist (NF157) for 30 min, then treated with 10 μM Ap_4_A for 2 h (B), and analyzed for *IL8* (A + B) and *TNFA* (C) levels using RT–qPCR. The *IL8* and *TNFA* levels were normalized to *GAPDH* and the mean ± SD of three biological replicates shown. ****P < 0.0001, ***P < 0.001, *P < 0.05 (two‐way ANOVA with Dunnett's (A + C) or Šídák's (B) multiple comparison test). The data presented are a representative of two (A + C) and three (B) independent experiments.DDifferentiated BLaER1 NTC and *P2RY11* KO cells treated with 500 μM ATP for 10 min followed by western blotting against phosphorylated and total ERK1/2 and p38 and β‐actin.E–HTranscriptomic analysis of differentiated BLaER1 NTC and *P2RY11* knockout cells treated with 500 μM Ap_4_A, ATP, or ADP for 2 h. Principal component analysis (PCA) on global gene expression profiles of individual biological replicates. Colors represent different treatment groups, shapes represent the cell line, and axes represent the first two components (E). The number of distinct transcripts that are significantly regulated (adjusted *P*‐value ≤ 0.01, log2 fold change ≥ 1) in *P2RY11* KO cells relative to NTC cells after treatment (F). Gene set enrichment analysis of the regulated transcripts from (F) using Reactome database (Fisher's exact test unadjusted *P*‐value ≤ 0.001) (G). Heat map of the log2 fold change gene expression (KO vs. NTC within a nucleotide treatment) for transcripts within the interleukin signaling, NGF transcription factor, and RAF‐independent MAPK1‐3 signaling terms from (G) (H). Source data are available online for this figure.

In order to link the inflammatory functions of the adenine‐derived nucleotides to endogenous P2YRs, we depleted the individual P2YRs in BLaER1 cells. The *P2RY* knockout cells were differentiated into macrophages and stimulated with Ap_4_A, ATP, or ADP for 2 h. mRNA expression levels of targeted P2YRs in the individual differentiated BLaER1 knockout cells indicated at least partial gene deletion for the detectable P2Rs (Appendix Fig S5A). This analysis further confirmed that knockout of individual P2YRs did not affect expression of other P2YRs (Appendix Fig S5A). The depletion of the P2YRs did not affect cell viability (Appendix Fig S5B). In response to adenine‐derived nucleotides, only the lack of *P2RY11* led to a significant decrease in Ap_4_A‐, ATP‐, and ADP‐induced *IL8* expression with the most prominent change being in the response to Ap_4_A (Fig 7A). We confirmed these data using the P2Y_11_ inhibitor NF157. In line with the P2Y_11_ knockout data, NF157 reduced *IL8* expression in wt BLaER1 macrophages stimulated with Ap_4_A (Fig 7B; Ullmann *et al*, 2005). Notably, P2Y_11_ was also responsible for the downregulation of *TNFA*, as treatment with ATP, ADP, or Ap_4_A led to an upregulation of *TNFA* mRNA in P2Y_11_‐depleted cells relative to nontargeting control cells (NTC; Fig 7C). The P2R Pan inhibitor PPADS targets a broad selection of P2YRs with the exception of P2Y_11_ (Communi *et al*, 1999). Notably, PPADS had no effect on the transcriptional regulation of adenine‐derived nucleotides, further supporting a prominent role of P2Y_11_ in sensing these nucleotides (Appendix Fig S6A).

The sensitivity of P2Y_11_ toward all adenine‐derived nucleotides led us to consider that degradation products, rather than the nucleotides themselves, are recognized by this receptor. However, differentiated BLaER1 cells exposed to the noncleavable ATP analog ATPγS displayed a transcriptional response that was comparable to the response seen for Ap_4_A and ATP and was similarly P2Y_11_ dependent (Appendix Fig S6B). Moreover, AMP and adenosine (ADO) were less potent in stimulating BLaER1 cells as compared to Ap_4_A, ATP, or ADP. Importantly, the AMP‐ and ADO‐driven response was P2Y_11_ independent (Appendix Fig S6C), thus excluding that P2Y_11_ detects adenine nucleotide‐derived degradation products.

Given the effect of P2Y_11_ in nucleotide‐driven cytokine responses, we tested whether this protein is regulating MAPK signaling in response to ATP stimulation. Indeed, while p38 was phosphorylated in NTC cells stimulated with ATP for 10 min, the same treatment did not induce prominent p38 phosphorylation in *P2RY11* knockout cells. Interestingly, ATP induced comparable phosphorylation of ERK1/2 in NTC and *P2RY11* knockout cells (Fig 7D). These data indicate that the ability to elicit p38 activation requires P2Y_11_, while the activation of the ERK signaling pathway is independent of P2Y_11_.

To better comprehend the function of P2Y_11_ in nucleotide sensing, we performed transcriptome analysis of NTC and *P2RY11* knockout cells that were treated with Ap_4_A, ATP, and ADP for 2 h (Datasets EV4 and EV5). PCA analysis showed a clear segregation of NTC and *P2RY11* knockout cells stimulated with nucleotides (Fig 7E). Directly comparing the number of regulated genes in the *P2RY11* knockout vs. NTC cells highlights the substantial transcriptional changes appearing upon depletion of *P2RY11* (Fig 7F). When comparing features of the differentially expressed genes between the *P2RY11* knockout and NTC by Reactome pathway enrichment analysis, the significantly enriched terms interleukin signaling, NGF‐stimulated transcription and RAF‐independent MAPK1‐3 signaling highlighted in Figs 3A, and 6A and B, reappear in the *P2RY11* knockout vs. NTC comparison (Fig 7G). Taking a closer look at the regulated genes in the knockout vs. NTC cells within these terms, P2Y_11_ seem to control the expression of all the essential genes regulated by the adenine‐derived nucleotide treatment. *IL8*, *IL1B*, *IL10*, *FOS*, *FOSB*, and *JUNB*, which were upregulated by the adenine‐derived nucleotides, were now downregulated in the knockout cells upon treatment. Reversely, *TNFA* and *CCL2* were upregulated in the knockout compared with the NTC macrophages (Fig 7H). This underlines that P2Y_11_ is responsible for the overall transcriptional regulation induced by the adenine‐derived nucleotides.

The knockout and small molecule inhibition experiments show that P2Y_11_ is central for the unique cytokine induction pattern caused by nucleotide treatments of the BLaER1 macrophages.

### A subset of P2YRs attenuate virus propagation in human macrophages

**Figure 8:**
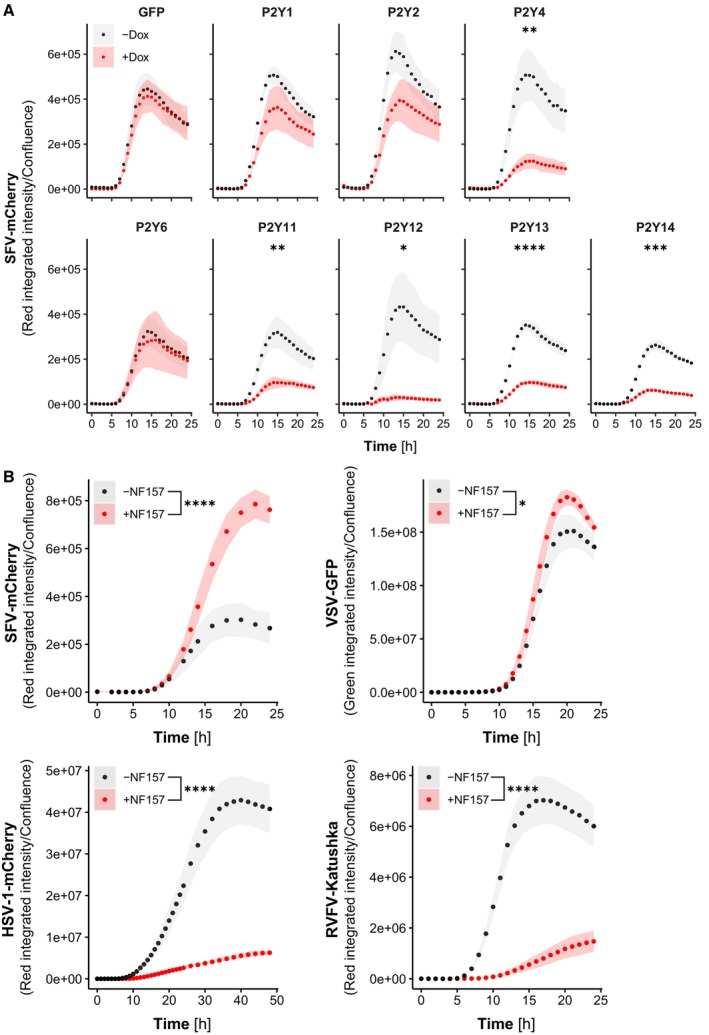
P2YRs suppress SFV infection A, BStable THP‐1 P2YR cells differentiated with 100 ng/ml PMA either with or without 1 μg/ml doxycycline for 24 h and then infected with SFV‐2SG‐mCherry (MOI 2) (A). THP‐1 cells differentiated with 100 ng/ml PMA for 24 h and then pretreated with 100 μM NF157 for 30 min prior to infection with SFV‐mCherry (MOI 2), RVFV‐Katushka (MOI 0.5), HSV‐1‐mCherry (MOI 1), or VSV‐GFP (MOI 0.5) (B). The fluorescent signal and cell confluence were tracked for 24 or 48 hpi using an IncuCyte S3 live imagining platform. The data presented are a representative of three independent experiments, and for each line diagram, the mean ± SD were calculated based on three (A) or four (B) biological replicates. ****P < 0.0001, ***P < 0.001, **P < 0.01, *P < 0.05 (unpaired two‐tailed *t*‐test at 15 hpi (A) or 20 hpi SFV‐mCherry, 20 hpi VSV‐GFP, 40 hpi HSV‐1‐mCherry, and 16 hpi RVFV‐RFP (B)).

The involvement of P2YRs in cytokine responses warrants the hypothesis that P2YRs also could be involved in restricting viral infections. The expression of P2Y_6_ and P2Y_14_ was upregulated by IFN‐α treatment (Fig 1B and Appendix Fig S1), indicating that the cytokine induction downstream of, for example, a viral infection could similarly regulate *P2RY* mRNA expression. Thus, differentiated BLaER1 cells were infected with HSV‐1, RVFV, IAV ΔNs1, SFV, and VSV‐M2 and analyzed for changes in the *P2RY* expression levels. The upregulation of *P2RY6* and *P2RY14* was common across viruses, while especially RVFV and IAV ΔNs1 seemed to have a broader effect on the *P2RY* expression (Appendix Fig S7). To systematically characterize the involvement of the P2YRs in viral infections, we generated THP‐1 cells expressing individual V5‐tagged P2YRs in a doxycycline‐inducible manner. The expression of the individual P2YRs and the control GFP upon doxycycline treatment was verified by western blotting (Appendix Fig S8A) and RT–qPCR (Appendix Fig S8B). The THP‐1 cells were differentiated into macrophages, and the expression of P2YRs was induced for 24 h. The overexpression of the individual P2YRs did not affect the endogenous mRNA levels of the other P2YR family members or the viability of THP‐1 macrophages (Appendix Fig S8B and C). In order to test whether the inducible expression of P2YRs affects virus growth, we infected differentiated THP‐1 cells with a recombinant Semliki forest reporter virus expressing mCherry (SFV‐mCherry) and used live‐cell fluorescence microscopy to follow the red fluorescence expression over time (Fig 8A). Comparison of the doxycycline‐treated and untreated GFP control cells indicated that expression of exogenous protein did not affect the SFV‐mCherry signal. Similar to GFP, doxycycline‐induced expression of P2Y_1_, P2Y_2_, and P2Y_6_ did not lead to differences in virus growth, as compared to untreated cells. Notably, however, doxycycline treatment of THP‐1 macrophages expressing P2Y_4_, P2Y_11_, P2Y_12_, P2Y_13_, and P2Y_14_ mitigated SFV replication, while mock‐treated cells showed accumulation of mCherry signal that was comparable to the accumulation in GFP‐expressing cells (Fig 7A and Appendix Fig S9). Collectively, this shows that a broad spectrum of P2YRs have the capability to reduce virus growth and highlights their involvement in antiviral immune responses.

Interestingly, receptors that are capable of restricting SFV replication (P2Y_4_, P2Y_11_, P2Y_12_, P2Y_13_, and P2Y_14_) seem to be distinct from the P2YRs capable of activating AP‐1 signaling (P2Y_1_, P2Y_2_, P2Y_6_, and P2Y_11_), suggesting that the two features are independent. Strikingly, P2Y_11_ was the only receptor capable of both inducing inflammatory responses and restricting replication of SFV. To investigate whether endogenous P2Y_11_ is involved in antiviral restriction in macrophages, we treated THP‐1‐derived macrophages with the P2Y_11_ antagonist NF157 prior to infection with SFV‐mCherry, RVFV‐Katushka, VSV‐GFP, and HSV‐1‐mCherry. The inhibition of P2Y_11_ significantly increased the replication of SFV and VSV, while the HSV‐1 and RVFV replication was significantly decreased (Fig 8B). This confirms that endogenous P2Y_11_ has an effect on viral infection in macrophages and that the specific effect depends on the virus. Overall, it highlights the importance of P2Y_11_ in macrophage immunity.

## Discussion

Studies of individual P2YRs have revealed these receptors as being important for various immune cell functions in both physiological and pathophysiological conditions (Jacob *et al*, 2013; Antonioli *et al*, 2019). Here, we studied the entire P2YR family in both inflammatory and infectious settings in human macrophages. Based on the P2YR trans‐complementation experiments, we found that the P2YRs can be divided into two distinct functional groups: an inflammatory group and an antiviral group. P2Y_4_, P2Y_12_, P2Y_13_, and P2Y_14_ have antiviral potential, which is independent of the ability to induce cytokines. In contrast, P2Y_1_, P2Y_2_, and P2Y_6_ have the capability to activate the AP‐1 promoter and ultimately cause the induction of multiple cytokines; however, they do not possess inherent antiviral properties. In macrophages, P2Y_11_ is the only receptor that is capable of both inducing an inflammatory response and regulating viral replication and thus has the potential of linking the two functions (Figs 6C and 8A). Indeed, we found that in macrophages P2Y_11_ is at least partially responsible for the inflammatory cytokine signature in response to adenine‐derived nucleotides and that inhibition of endogenous P2Y_11_ increases SFV replication (Figs 7 and 8B). Surprisingly, depletion of P2Y_11_ reduced HSV‐1 and RVFV infection, indicating that P2Y_11_ serves as a critical host factor for a subset of viruses. Although the underlying mechanisms are currently not clear, one could envision that P2Y_11_ promotes viral uptake through regulating the endocytosis machinery as has been proposed for P2Y_2_, which has the ability to regulate αV integrins (Erb *et al*, 2001; Bagchi *et al*, 2005; Tomas Bort *et al*, 2023). Alternatively, P2Y_11_ signaling may promote virus growth through activation of MAPK signaling, which is critical for HSV‐1 replication (Jang *et al*, 1991; McLean & Bachenheimer, 1999).

Transcriptome analysis showed that macrophages stimulated with adenine‐derived nucleotides produce a specific inflammatory response that we linked to MAPK activation of the AP‐1 transcription factors. P2Y_1_, P2Y_2_, P2Y_6_, and P2Y_11_ all have the capability to activate the AP‐1 promoter in response to adenine‐derived nucleotides (Fig 6C). The receptors P2Y_1_ and P2Y_11_ have been reported to have the highest affinity for ADP and ATP, respectively (Communi *et al*, 1997; Palmer *et al*, 1998), which fits with the here‐described preferential signaling in response to adenine‐derived nucleotides. P2Y_2_ has equally high affinities for ATP and UTP (Nicholas *et al*, 1996), which would explain why P2Y_2_ is able to elicit a minor activation of AP‐1 in response to UTP, in addition to adenine‐derived nucleotides. P2Y_6_ is known to be a UDP receptor (Communi *et al*, 1996) and the affinity for adenine‐containing nucleotides and the lacking response to UDP is surprising. However, a possible explanation may be that the selective expression of exogenous P2Y_6_ may alter selectivity toward adenine vs uridine nucleotides (Fig 6C). P2Y_1_, P2Y_2_, P2Y_4_, P2Y_6_, and P2Y_11_ are predominantly G_q_‐coupled receptors, while P2Y_12_, P2Y_13_, and P2Y_14_ are exclusively G_i_‐coupled receptors (Erb & Weisman, 2012), suggesting that the activation of the AP‐1 promoter could be accredited to G_q_ activation. G_q_‐coupled receptors activate the phospholipase C (PLC) pathway as the major GPCR pathway, which is known to activate several MAPKs through activation of PKC (Goldsmith & Dhanasekaran, 2007). An involvement of the MAPK pathway in response to nucleotide treatment in macrophages is supported by the nucleotide‐dependent phosphorylation of the MAPKs ERK1/2 and p38 (Figs 6D and 7D) and transcriptome data revealing differential regulation of DUSP genes known for the regulated dephosphorylation and inactivation of MAPK family members (Fig 6B).

The reason for the importance of P2Y_11_ in the macrophage cytokine responses to adenine‐derived nucleotides may lie in the cell‐specific P2YR expression pattern and ligand specificity. The ATP/UTP receptor P2Y_2_ is poorly expressed at mRNA level and is most likely not substantially expressed as protein on the cell surface of the BLaER1 macrophages. Despite the ability of P2Y_6_ to respond to adenine‐derived nucleotides in our overexpression setting, P2Y_6_ is indeed a high‐affinity UDP receptor and since UDP is not able to induce *IL8* mRNA expression in the macrophages, it may explain why P2Y_6_ does not have a major contribution in the macrophage inflammation in response to adenine‐derived nucleotides. P2Y_11_ and P2Y_1_ are known to interact and form a hetero‐oligomer, which changes the signaling profile of the two receptors; thus, it is possible that in macrophages P2Y_11_ influences P2Y_1_ or vice versa (Ecke *et al*, 2008). P2Y_1_ and P2Y_11_ share the ability to activate G_q_, but P2Y_11_ is the only P2YR that in addition to G_q_ can couple to G_s_ (Communi *et al*, 1997; Qi *et al*, 2001), which activates adenylate cyclase (ADCY) resulting in a rise in intracellular cAMP. Elevated cAMP has been demonstrated to inhibit NF‐κB transcription (Takahashi *et al*, 2002; Minguet *et al*, 2005; Gerlo *et al*, 2011), which could explain the P2Y_11_‐dependent inhibition of basal *TNFA* expression. The induction of *IL8* in response to nucleotides is NF‐κB independent (Fig 5A) and would therefore not be affected by the elevation of cAMP. In fact, we observe that ATP stimulation of macrophages actually downregulates the basal phosphorylation of p65 (Fig 5B), a component of NF‐κB, supporting the inhibitory function. We thus hypothesize that the dual G protein coupling of P2Y_11_ is responsible for the specific cytokine expression pattern in response to adenine‐derived nucleotides.

A pro‐inflammatory role of P2Y_11_ is supported by data on patients with rheumatoid arthritis (RA), who show elevated expression of P2Y_11_ in their fibroblast‐like synoviocytes as compared to healthy individuals. Chronic inflammation of the joints plays an important role in the development of RA, and the synoviocytes play a key role in producing pro‐inflammatory cytokines such as IL‐1β. Suppression of P2Y_11_ with an antagonist prevents the activation of NF‐κB by IL‐1β in synoviocytes and the downstream expression of additional pro‐inflammatory cytokines such as TNF‐α and IL‐6 (Gao & Li, 2019). This notion is in line with our finding that P2Y_11_ can induce an inflammatory response and that this receptor has the capability to modulate the local inflammatory environment toward pro‐inflammatory signaling in specific cases.

As opposed to P2Y_11_, the P2Y_4_, P2Y_12_, P2Y_13_, and P2Y_14_ receptors all have the potential to be antiviral against SFV in macrophages, which is seemingly independent of classical cytokine production. Except for P2Y_4_, the P2YRs with exclusive antiviral activity are G_i_‐coupled receptors (Erb & Weisman, 2012), and thus, the antiviral activity of these P2YRs could originate from G_i_ activation. G_i_ activation inhibits ADCY and thus negatively regulates the formation of cAMP. In cases of cAMP elevation by ADCY, cAMP is detected by cAMP receptors such as protein kinase A (PKA) and exchange proteins directly activated by cAMP (EPAC; Robichaux III & Cheng, 2018). Notably, a role of EPAC has been shown for infection with several viral pathogens. Ebola virus, MERS‐corona virus and respiratory syncytial virus all activate EPAC‐mediated pathways to favor virus invasion (Tao *et al*, 2014; Choi *et al*, 2018; Drelich *et al*, 2018; Ren *et al*, 2021). In addition, P2Y_13_‐mediated inhibition of cAMP has been directly linked to viral restriction of vesicular stomatitis virus (VSV) through inhibited EPAC1 activation (Zhang *et al*, 2019), suggesting that the antiviral potential of the overexpressed receptors could be mediated through inhibiting viral invasion. The effect on viral invasion could also explain the independence of the production of pro‐inflammatory cytokines in the antiviral effect in the macrophages. As opposed to P2Y_12_, P2Y_13_ and P2Y_14_, P2Y_11_ activates ADCY through G_s_ and increases intracellular cAMP, but is similarly presenting with antiviral properties against SFV. P2Y_11_ has, in overexpression studies, shown to be more potent in the activation of PLC through G_q_ than activation of ADCY through G_s_ (Qi *et al*, 2001), which may indicate that the production of cytokines downstream of G_q_ activation and the antiviral effect of these cytokines are dominant over the potential pro‐viral effect of G_s_ activation of ADCY‐dependent cAMP elevation.

In conclusion, we investigated the P2Ys for their ability to induce inflammatory responses and to inhibit virus infection. Collectively, our study reveals a surprising complexity of P2YR signaling, governed by cell‐specific expression and individual activity patterns of P2YRs, which in sum has the propensity to regulate inflammatory and antiviral responses.

## Materials and Methods

### Cell culture, treatments, and viruses

HEK293‐R1, A549, Huh7.5, HeLa, HeLa Kyoto, SKN‐BE2, HEK293T, and HEK293T‐TLR4‐CD14‐MD2 (Prof. Melanie M Brinkmann) cells were cultured in DMEM supplemented with 10% (v/v) FCS and 1% penicillin/streptomycin (P/S). BLaER1 and THP‐1 cells were cultured in RPMI‐1640 medium supplemented with 10% (v/v) FCS and 1% (v/v) P/S. For cultivating BLaER1 cells, the medium was additionally supplemented with 1 mM sodium pyruvate (Media constituents from Sigma‐Aldrich). BLaER1 cells were transdifferentiated into macrophages for 6 days in growth medium containing 10 ng/ml human IL‐3 (PeproTech 200‐03), 10 ng/ml human M‐CSF (PeproTech 300‐25), and 100 nM β‐estradiol (Sigma‐Aldrich E2758) at 7.5 × 10^5^ cells per 12 well. THP‐1 cells were differentiated with 100 ng/ml phorbol 12‐myristate 13‐acetate (PMA; Sigma‐Aldrich P1585) at 7.5 × 10^5^ cells per 96 well for live‐cell imaging or 1 × 10^6^ cells per 12 well for RT–qPCR for 24 and 48 h, respectively.

The cells were treated with Ap_4_A (Sigma‐Aldrich D1262, Jena Bioscience NU‐507), ATP (Sigma‐Aldrich A6419, Jena Bioscience NU‐1198), ATPγS (Jena Bioscience NU‐405), ADP (Sigma‐Aldrich A2754, Jena Bioscience NU‐1010), AMP (Jena Bioscience NU‐1025), Adenosine (Sigma‐Aldrich A4036), UTP (Sigma‐Aldrich U6750, Jena Bioscience NU‐1206), UDP (Sigma‐Aldrich 94330, Jena Bioscience NU‐1013), IFN‐α B/D (Prof. Dr. Peter Stäheli), LPS (Sigma‐Aldrich L2630), NF157 (Tocris Bioscience 2450), A438079 (Tocris Bioscience 2972), and PPADS (Tocris Bioscience 0625). The concentration and treatment time used are indicated for the individual experiments.

The cells were infected with SFV (Prof. Georg Kochs), VSV‐M2, IAV S35M ΔNs1, HSV‐1, RVFV clone 13, SFV‐2SG‐mCherry (Prof. Andres Merits), RVFV‐Katushka (RFP; Prof. Friedemann Weber), HSV‐1(17+)Lox‐mCherry (Prof. Beate Sodeik), or VSV‐GFP. The MOI and infection time used are indicated for the individual experiments.

### Generation of stable P2YR overexpression THP‐1 cell lines

cDNA for the eight P2YRs was cloned into the pLIX304‐V5 vector. Lentiviral particles were generated by transient transfection of HEK293T cells with pLIX304‐V5, pMD2‐VSVG, and pCMV‐Gag‐Pol (6/2.1/3.9 μg) vectors using a polyethylenimine (PEI):DNA ratio of 3:1. 48‐h post‐transfection viral supernatants were collected, filtered through a 0.45 μm filter, and stored at −80°C until further use. The lentiviral titer was determined as the colony‐forming units in infected HeLa Kyoto cells. THP‐1 cells were seeded at 2 × 10^5^ cells/ml and transduced with lentivirus (MOI 1) and 24‐h post‐transduction put under selection with 1 μg/ml puromycin (Sigma P8833) to select for P2YR‐containing cells.

### Generation of BLaER1 KO cells

For the P2YR family target genes, polyclonal knockout cells were generated. To this end, CRISPR–Cas9–RNPs were assembled by annealing synthetic, chemically stabilized crRNA:tracrRNA pairs (IDT) at 95°C for 5 min, and incubated at room temperature for 30 min. For every target gene, two gRNAs were used (Dataset EV6). For every gRNA pair, 40 pmol Cas9 was added for each 100 pmol of gRNA and this mixture was incubated for 10 min at room temperature. For nucleofection, 1 million BLaER1 cells were resuspended in 20 μl of nucleofection buffer SF (Lonza). This buffer was supplemented with RNPs and nucleofected with the program CA‐137. After nucleofection, cells were collected from the nucleofection cuvettes with warm medium and transferred to a 6‐well plate. Cells were rested for 48 h and used as pool knockouts.

The other BLaER1 knockout cells were generated as clonal knockout cells. To this end, sgRNA oligos were designed as previously described (Schmidt *et al*, 2015) or using CHOPCHOP (Labun *et al*, 2019) and cloned into expression plasmids (Schmid‐Burgk *et al*, 2014; Schmidt *et al*, 2015; Dataset EV6). BLaER1 cells were electroporated with plasmids driving expression of Cas9 and a sgRNA on a Bio‐Rad GenePulser XCell. Following electroporation, cells were then subjected to single‐cell cloning. After 3–4 weeks, thus‐derived monoclones were analyzed by Illumina sequencing as described previously (Schmid‐Burgk *et al*, 2014; Schmidt *et al*, 2015). Monoclones with all‐allelic frame‐shifting mutations were then used as knockout cell clones.

### Live cell imaging of viral replication

The stable P2YR THP‐1 cells were differentiated with PMA and treated with 1 μg/ml doxycycline to induce expression of the P2YRs for 24 h. The cells were then infected with SFV‐2SG‐mCherry (MOI 2), RVFV‐Katushka (RFP; MOI 0.5), HSV‐1(17+)Lox‐mCherry (MOI 1) or VSV‐GFP (MOI 0.5). Fluorescence intensity was measured every 1 for 24 h using an IncuCyte S3 fluorescence light microscopy screening platform (Sartorius). The fluorescence intensity of the reporter viruses was assessed as integrated intensity per image normalized to cell confluence per image using IncuCyte S3 Software.

### Promoter luciferase reporter assays

HEK293‐R1 or HEK293T‐TLR4‐CD14‐MD2 cells were seeded at 3 × 10^5^ cells per 24 well for 24 h. The HEK293‐R1 cells were transfected with 100 ng pAP‐1‐Luc, 12 ng pBS‐EIF1a‐Ren, and 200 ng pLIX403 encoding V5‐tagged GFP, P2Y_1_, P2Y_2_, P2Y_4_, P2Y_6_, P2Y_11_, P2Y_12_, P2Y_13_, or P2Y_14_, while the HEK293T‐TLR4 cells were transfected with 100 ng pNF‐kB‐Luc and 12 ng pBS‐EIF1a‐Ren using 1 μl METAFECTENE Pro (Biontex) for both cell lines. The pLIX403‐V5 vector contains a Tet operator, and thus, the HEK293‐R1 cells were upon transfection stimulated with 1 μg/ml doxycycline for 8 h. After 8 h, the cells were stimulated with 500 μM ATP, ADP, Ap_4_A, UTP, or UDP for 16 h. The transfected HEK293T‐TLR4 cells were incubated for 16‐h post‐transfection and stimulated with 500 μM ATP, ADP, Ap_4_A, UTP, UDP, or various concentrations of LPS for 8 h. The Firefly and *Renilla* Luciferase activities were measured with the Dual‐Luciferase® Reporter Assay System (Promega E1960) according to the manufacturer's protocol using the Infinite® 200 PRO series microplate reader (TECAN).

### Flow cytometry

Research blood samples were obtained from leucocyte reduction chambers from thrombocyte donations (LMU Klinikum, Munich, Germany, ethics committee, no. 18‐415). Peripheral blood mononuclear cells (PBMCs) were isolated by Ficoll density gradient centrifugation and cultured at density of 10^6^ cells/ml in RPMI 1640 (Biochrom, 10% FCS, 100 U/ml penicillin, 100 μg/ml streptomycin, 1% nonessential amino acids, 1 mM sodium pyruvate, 2 mM GlutaMAX, 0.05 mM β‐mercaptoethanol). The PBMCs were stimulated for 8 h with Ap_4_A (500 μM) or LPS (10 ng/ml). After the initial 4 h, GolgiPlug™ (BD, #555029) and GolgiStop™ (BD, #554724) were added according to the manufacturer's instructions. A fixable viability dye was used according to the manufacturer's protocol, and the cells were stained for surface markers in 100 μl of PBS, 2 mM EDTA, 10% FCS (v/v) containing FcR blocking reagent (Miltenyi Biotec) with fluorescently labeled antibodies for 30 min at 4°C. The intracellular staining for IL‐8 was performed using the Transcription Factor Staining Buffer Set (Thermo Fisher, #00‐5523‐00) following the manufacturer's instructions. The following antibodies were used: CD56 (BioLegend, clone 5.1H11, 1 to 40), CD8 (BioLegend, clone SK1, 1:100), HLA DR (BioLegend, clone L243, 1:50), CD11c (BioLegend, clone 3.9, 1:100), CD19 (Biolegend, HIB19, 1:100), CD3 (eBioscience, clone OKT3, 1:100), CD123 (eBioscience, clone 7G3, 1:100), IL‐8 (BioLegend, clone E8N1, 1:100), CD14 (BioLegend, clone M5E2, 1:200), CD4 (eBioscience, clone OKT4, 1:100), CD16 (BioLegend, clone 3G8, 1:100), and Zombie Red™ Fixable Viability Kit. Samples were measured using Cytek Aurora (Cytec Biosciences).

### Cell viability assay

The cell viability was determined by quantifying the amount of ATP present in the cells using the CellTiter‐Glo® Luminescent Cell Viability Assay (Promega #G7570). The media was first aspirated from the cells. Suspension cells were resuspended, collected in tubes, and centrifuged prior to media aspiration. The cells were lysed in 70 μl CellTiter‐Glo® Reagent and 20 μl lysates transferred to a white 96‐well round bottom plate in triplicate. The luminescence was measured with the Tecan plate reader.

### Transcriptomic sample preparation and measurement

Total RNA was extracted from BLaER1 macrophages stimulated with various nucleotides for different times using the NucleoSpin® RNA Plus kit (Macherey‐Nagel) according to the manufacturers' protocol.

Library preparation for bulk 3′‐sequencing of poly(A)‐RNA was done as detailed in Parekh *et al* (2016)). Briefly, barcoded cDNA of each sample was generated with a Maxima RT polymerase (Thermo Fisher) using oligo‐dT primer containing barcodes, unique molecular identifiers (UMIs), and an adapter. 5′ ends of the cDNAs were extended by a template switch oligo (TSO), and after pooling of all samples, full‐length cDNA was amplified with primers binding to the TSO site and the adapter. cDNA was fragmented and TruSeq‐Adapters ligated with the NEBNext® Ultra™ II FS DNA Library Prep Kit for Illumina® (NEB), and 3′‐end fragments were finally amplified using primers with Illumina P5 and P7 overhangs. In comparison with Parekh *et al*, the P5 and P7 sites were exchanged to allow sequencing of the cDNA in read1 and barcodes and UMIs in read2 to achieve better cluster recognition. The library was sequenced on a NextSeq 500 (Illumina) with 75 cycles for the cDNA in read1 and 16 cycles for the barcodes and UMIs in read2.

### Transcriptomic analysis

For RNA‐seq data analysis, Gencode gene annotations v35 and the human reference genome GRCh38 were derived from the Gencode homepage (EMBL‐EBI). Dropseq tools v1.12 was used for mapping raw sequencing data to the reference genome (Macosko *et al*, 2015). The subsequent normalization and differential expression analysis was performed by DESeq2 package (version 1.34.0; Love *et al*, 2014) using the following linear model in R notation for the nucleotide stimulation time course dataset (Fig 2):

$$\log_{2}\left(\text{gene expression}\;\left(t\right)\right)\sim \sum_{t_{i}\leq t}\left(\text{after}\;\left(t_{i}\right)+\text{treatment}:\text{after}\;\left(t_{i}\right)\right)$$

where the after (*t*_*i*_) effect represents the change of gene expression in mock‐treated samples between *t*_*i*−1_ and *t*_*i*_ post‐treatment and applies to the modeled gene expression at all time points since *t*_*i*_; treatment:after (*t*_*i*_) is the specific effect of treatment (Ap_4_A, ATP, ADP, UTP, and UDP) occurring between *t*_*i*−1_ and *t*_*i*_ post‐treatment.

Similarly, the following linear model was used for the *P2RY11* knockout dataset (Fig 7):

$$\log_{2}\left(\text{gene expression}\right)\sim \text{knockout}*\text{treatment}$$

where knockout represents whether *P2RY11* was knocked out in the cells. Treatment included Ap_4_A, ATP, and ADP.

The log_2_ fold changes were then shrunken via ashr (Stephens, 2017). A gene was considered significantly changing in comparison with mock at a given time if the absolute shrunken log_2_ fold change was at least 1 and the adjusted *P*‐value was at most 0.01. The PCA was performed by the built‐in function of DESeq2 on variance‐stabilizing transformed counts.

We used the Enrichment Map gene sets (version 2021.12) of human proteins that included Gene Ontology and Reactome databases for the enrichment analysis (Merico *et al*, 2010). To minimize the redundancy in the enriched terms, we employed our in‐house Julia package OptEnrichedSetCover.jl (https://doi.org/10.5281/zenodo.4536596) to identify the collection of terms with minimal pairwise overlaps and significant enrichment in individual comparisons. The outcome was further filtered by having an unadjusted *P*‐value smaller or equal to 0.001 (Fisher's exact test) in at least one comparison. Unless otherwise stated, the analysis was done in R (version 4.1) and Julia (version 1.6) with in‐house scripts.

### RT–qPCR analysis

Total RNA was extracted using the NucleoSpin® RNA Plus kit (Macherey‐Nagel) according to the manufacturers' protocol. Total RNA was used for reverse transcription with PrimeScript™ RT reagent Kit with gDNA Eraser (TaKaRa) according to the manufacturers' instructions. Relative transcript quantification was obtained by qPCR with the transcript‐specific primers (Dataset EV6) and PowerUp SYBR Green master mix (Thermo Fisher) on a QuantStudio3 Real‐time PCR system (Applied Biosystems). Ct values were obtained using QuantStudio Design and Analysis software and averaged across technical replicates, and the transcript levels were normalized to the levels of a housekeeping gene.

### Cytometric bead array (CBA)

Cytokine levels in the cell culture supernatants were assessed using the Bio‐Plex Pro Human Cytokine 17‐plex Assay (Bio‐Rad) according to the manufacturer's protocol and measured using a Bio‐Plex 200 Luminex Technology (Bio‐Rad).

### Western blotting

Cells were lysed in SDS lysis buffer (62.5 mM Tris–HCl pH 6.8, 50 mM Dithiothreitol (DTT), 10% Glycerol, 2% Sodium dodecyl sulfate (SDS), and 0.01% Bromophenol blue) supplemented with 1× PhosSTOP (Sigma‐Aldrich) and 1× cOmplete™, EDTA‐free Protease inhibitor cocktail (Sigma‐Aldrich). The lysates were sonicated using the Bioruptor (Diagenode) and treated with 750 U/ml DNase. The protein concentration was measured with the Pierce 660 reagent with added IDCR reagent (Thermo Fischer Scientific) according to the manufacturer's instructions, and the total protein concentration of the samples was equalized to the sample with the lowest concentration by dilution in lysis buffer.

Proteins for phospho‐western blot were separated on 4–12% Bis‐Tris gels (NuPAGE™, Thermo Fischer Scientific) and transferred to a 0.2 μm nitrocellulose membrane (Amersham™ Protran Premium, GE Healthcare), while proteins for P2YR‐V5 detection were separated on 12% polyacrylamide gels and transferred to a 0.45 μm PVDF membrane (Thermo Fisher Scientific). Membranes were incubated with primary antibodies at 4°C overnight and HRP‐conjugated secondary antibodies at room temperature for 1 h. The following antibodies were used: Horse Anti‐mouse IgG‐HRP (Cell Signaling Technology, 1:2,000), Goat Anti‐rabbit IgG‐HRP (Cell Signaling Technology, 1:5,000), Mouse Monoclonal Anti‐β‐Actin (C4) (Santa Cruz Biotechnology, 1:2,500), Mouse Monoclonal Anti‐V5‐HRP (V5‐10) (Sigma‐Aldrich, 1:2,000), Mouse Monoclonal Anti‐IκBα (L35A5) (Cell Signaling Technology, 1:1,000), Rabbit Monoclonal Anti‐Phospho‐IRF‐3 (Ser386) (E7J8G) (Cell Signaling Technology, 1:1,000), Rabbit Monoclonal Anti‐IRF‐3 (D6I4C) (Cell Signaling Technology, 1:1,000), Rabbit Monoclonal Anti‐Phospho‐NF‐κB p65 (Ser536) (93H1) (Cell Signaling Technology, 1:1,000), Rabbit Monoclonal Anti‐NF‐κB p65 (D14E12) (Cell Signaling Technology, 1:1,000), Mouse Monoclonal Anti‐Phospho‐p44/42 MAPK (Erk1) (Tyr204)/(Erk2) (Tyr187) (D1H6G) (Cell Signaling Technology, 1:1,000), and Rabbit Monoclonal p44/42 MAPK (Erk1/2) and (Cell Signaling Technology, 1:1,000). In between antibodies, the membranes were stripped with Restore Western Blot Stripping Buffer (Thermo Fischer Scientific). Immunoblots were developed with the SuperSignal™ West Femto kit (Thermo Fisher Scientific) and imaged with the Bio‐Rad ChemiDoc Imaging System.

## Author contributions

**Line Lykke Andersen:** Conceptualization; formal analysis; investigation; methodology; writing – original draft; project administration; writing – review and editing. **Yiqi Huang:** Formal analysis; visualization. **Christian Urban:** Formal analysis; investigation. **Lila Oubraham:** Investigation. **Elena Winheim:** Formal analysis; investigation; visualization. **Che Stafford:** Investigation. **Dennis Nagl:** Investigation. **Fionan O'Duill:** Investigation. **Thomas Ebert:** Investigation. **Thomas Engleitner:** Formal analysis. **Søren Riis Paludan:** Funding acquisition. **Anne Krug:** Supervision. **Roland Rad:** Supervision. **Veit Hornung:** Supervision; funding acquisition. **Andreas Pichlmair:** Conceptualization; funding acquisition; writing – original draft; project administration; writing – review and editing.

## Disclosure and competing interests statement

The authors declare that they have no conflict of interest.

## Supporting information



Appendix

Dataset EV1

Dataset EV2

Dataset EV3

Dataset EV4

Dataset EV5

Dataset EV6

Source Data for Figure 1

Source Data for Figure 3

Source Data for Figure 5

Source Data for Figure 6

Source Data for Figure 7

## Data Availability

The transcriptomic data are available on the ENA database under the accession PRJEB60753 (http://www.ebi.ac.uk/ena/data/view/PRJEB60753).
