# Supplementary material for: Systematic P2Y receptor survey identifies P2Y11 as modulator of immune responses and virus replication in macrophages
Source: EMBO J. 2023 Oct 26;42(23):e113279. doi: 10.15252/embj.2022113279 (PMC10690470; doi:10.15252/embj.2022113279)
Supplement: Supplementary file 1 — Appendix [file EMBJ-42-e113279-s009.pdf]

Table of Contents:

Appendix Figure S1: Interferon inducibility of P2RYs

Appendix Figure S2: Evaluating BlaER1 cell markers, ligand purity and toxicity

Appendix Figure S3: Nucleotide stimulate independently of TLR4 and P2X7

Appendix Figure S4: Cell viability in NF-kB knockout cells

Appendix Figure S5: P2RY knockout cell quality controls

Appendix Figure S6: Functional characterization of P2RY11 knockout cells

Appendix Figure S7: P2RY expression in virus infected cells

Appendix Figure S8: Inducible P2RY expression in THP-I cells

Appendix Figure S9: P2RY expression inhibits SFV growth

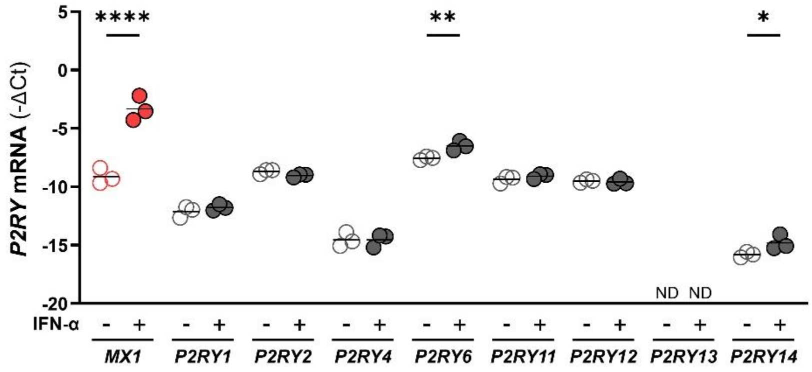

Appendix Figure S1: Interferon inducibility of P2RYs

THP-1 cells differentiated with 100 ng/mL PMA for 48 h, treated with 250 U/mL IFN-α for 8 h and analyzed for the endogenous *P2RY* levels by RT-qPCR. The P2RY levels were normalized to *GAPDH* and shown as the individual values of three independent experiments together with the mean and ****p < 0.0001, ***p < 0.001, **p < 0.01, *p < 0.05 (Two-way ANOVA with Šídák's multiple comparison test).

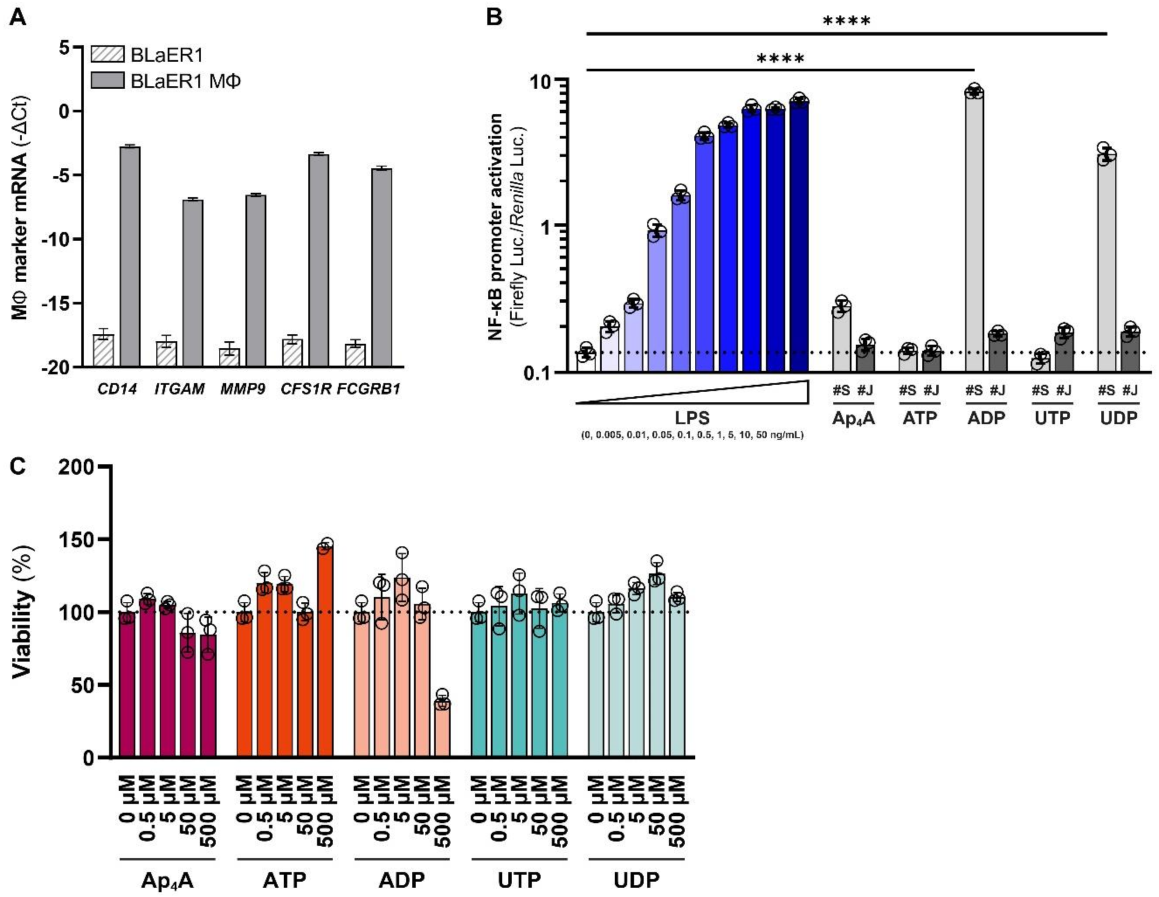

Appendix Figure S2: Evaluating BlaER1 cell markers, ligand purity and toxicity

(A) Total RNA from undifferentiated and differentiated BLaER1 cells analyzed for the levels of macrophage markers *CD14*, *ITGAM*, *MMP9*, *CFS1R* and *FCGRB1* by RT-qPCR and normalized to *GAPDH*. The data is shown as the mean ±SD of technical triplicates.

(B) HEK293T-TLR4-CD14-MD2 cells transfected with NF-κB promoter Firefly luciferase and EF-1α promoter *Renilla* reporter plasmids and after 16 h treated with 500 µM Ap_4A, ATP, ADP, UTP or UDP from two different manufacturers for 8 h. The data presented is a representative of three independent experiments and the graph shows the Firefly/*Renilla* signal from three biological replicates together with mean ± SD. ****$p < 0.0001$ (Two-way ANOVA with Dunnett's multiple comparison test, comparison to mock).

(C) Differentiated BLaER1 cells treated with various concentrations of Ap_4A, ATP, ADP, UTP or UDP for 2 h. ⅛ of the treated cells were used for the viability measurement using the CellTiter-Glo assay, while the rest was used for total RNA isolation and RT-qPCR analysis (Fig. 3C). The viability is expressed as the percentage of the mock average and the mean ±SD of three biological replicates shown. The data presented is a representative of two independent experiments.

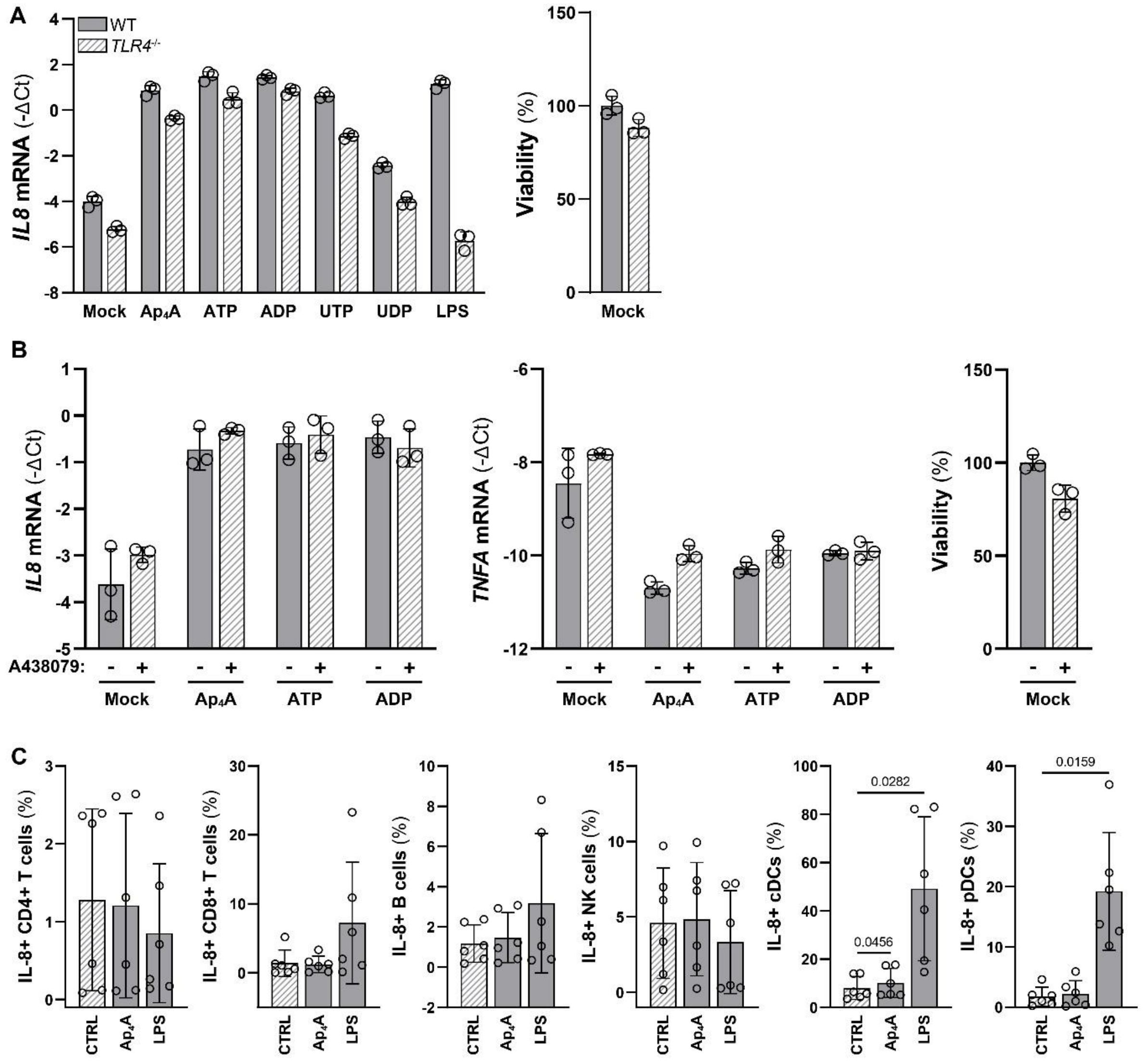

Appendix Figure S3: Nucleotide stimulate independently of TLR4 and P2X7

(A-B) Differentiated BLaER1 WT and TLR4 KO cells treated with 500 µM Ap_4A, ATP, ADP, UTP or UDP or 0.1 ng/mL LPS for 2 h (A) or differentiated BLaER1 WT cells pretreated with 10 µM $P2X_7$ antagonist (A438079) for 45 min, then treated with 500 µM Ap_4A, ATP or ADP for 2 h (B) and the levels of *IL8* and *TNFA* analyzed by RT-qPCR. The *IL8* and *TNFA* levels were normalized to *GAPDH* and the mean ±SD of three biological replicates shown. ⅛ of untreated cells were used for the viability measurement using the CellTiter-Glo assay. The viability is expressed as the percentage of the mock average and the mean ±SD of three biological replicates shown. The data presented is a representative of three independent experiments.

(C) PBMCs isolated from six donors treated with 500 µM Ap_4A or 10 ng/mL LPS for 8 h with protein secretion blocked after the initial 4 h. The cells were stained for viability using a fluorescent cell permeability dye and intracellular IL-8 and surface CD3 (T cells), CD4 (T cells), CD8 (T cells), CD56 (NK-cells), HLA-DR (Antigen presenting cells), CD11c (cDCs), CD19 (B cells) and CD123 (pDCs) using specific fluorescently coupled antibodies and analyzed by flow cytometry. The percentage of IL-8+ cells of the total immune cell subset is shown for each donor together with the mean ± SD and p-value (One-way repeated measures ANOVA with Dunnett's multiple comparison test).

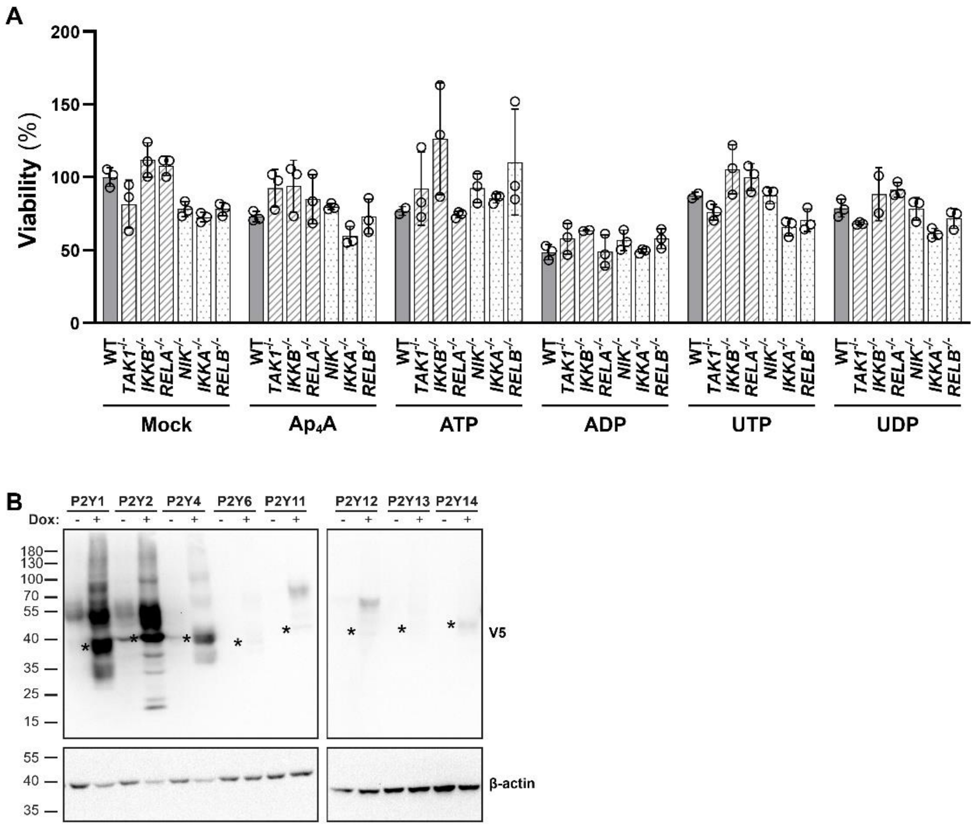

Appendix Figure S4: Cell viability in NF-kB knockout cells

(A) Differentiated BLaER1 WT or indicated KO cells treated with 500 µM Ap_4A, ATP, ADP, UTP or UDP for 2 h. ⅛ of the treated cells were used for the viability measurement using the CellTiter-Glo assay, while the rest was used for RNA isolation and RT-qPCR analysis shown in Fig. 5A. The viability is expressed as the percentage of the mock average and the mean ±SD of three biological replicates shown. The data presented is a representative of two independent experiments.

(B) Western blotting of V5-tagged P2YRs and β-actin in HEK293-R1 cells transiently transfected with the individual P2YRs with or without the presence of 1 µg/mL doxycycline.

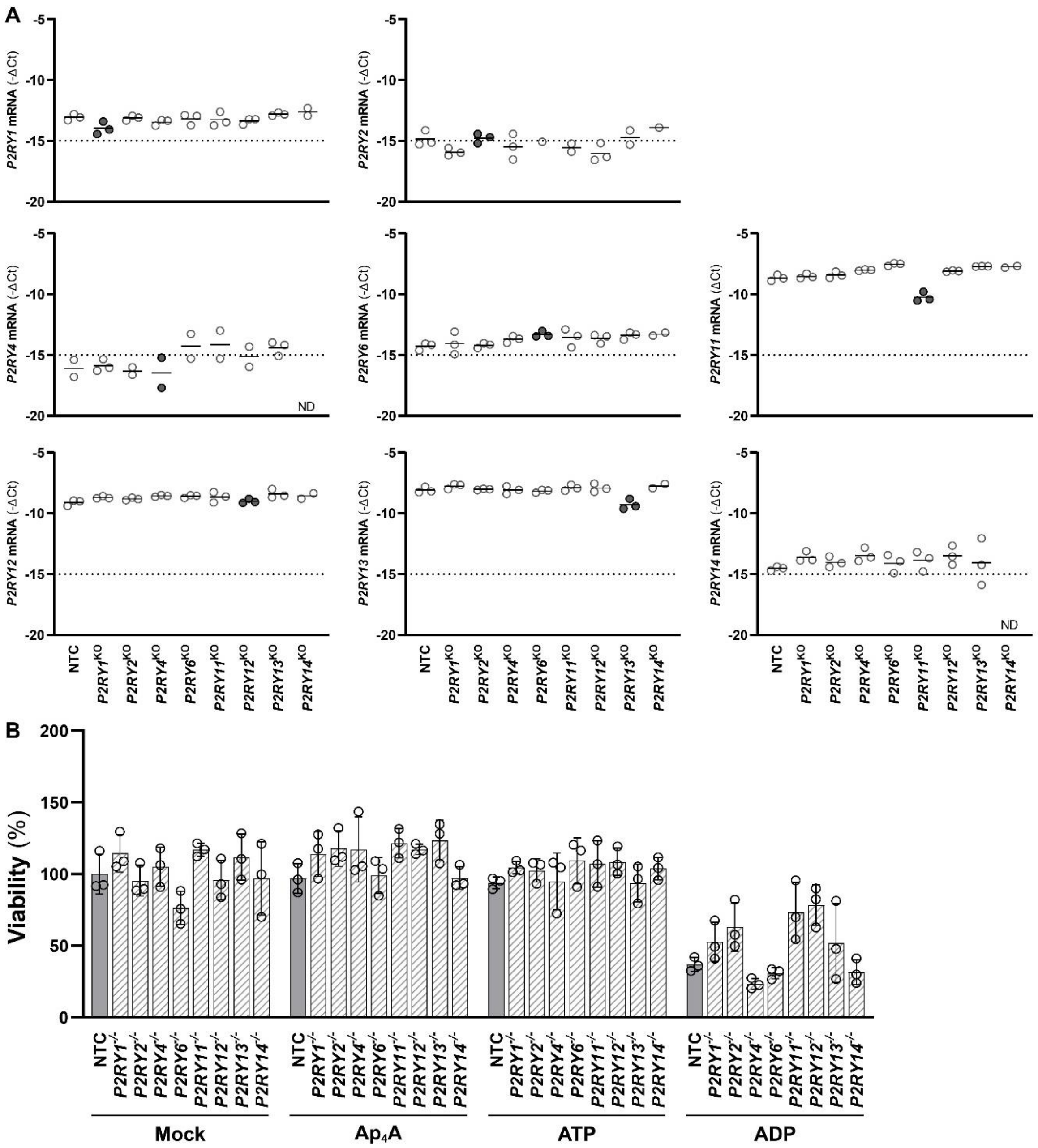

Appendix Figure S5: P2RY knockout cell quality controls

(A) Total RNA from differentiated BLaER1 NTC and P2RY KO cells were analyzed for the endogenous *P2RY* levels by RT-qPCR. The *P2RY* levels were normalized to *GAPDH* and the mean ±SD of three biological replicates shown. Missing dots are due to failure to detect the *P2RY* mRNA in all the replicates. The dotted line represents the subjectively judged confident detection limit of the RT-qPCR. **(B)** Differentiated BLaER1 WT or P2RY KO cells treated with 500 µM Ap_4A, ATP or ADP for 2 h. ⅛ of the treated cells were used for the viability measurement using the CellTiter-Glo assay, while the rest was used for RNA isolation and RT-qPCR analysis shown in Fig. 7A. The viability is expressed as the percentage of the mock average and the mean ±SD of three biological replicates shown. The data presented is a representative of two independent experiments.

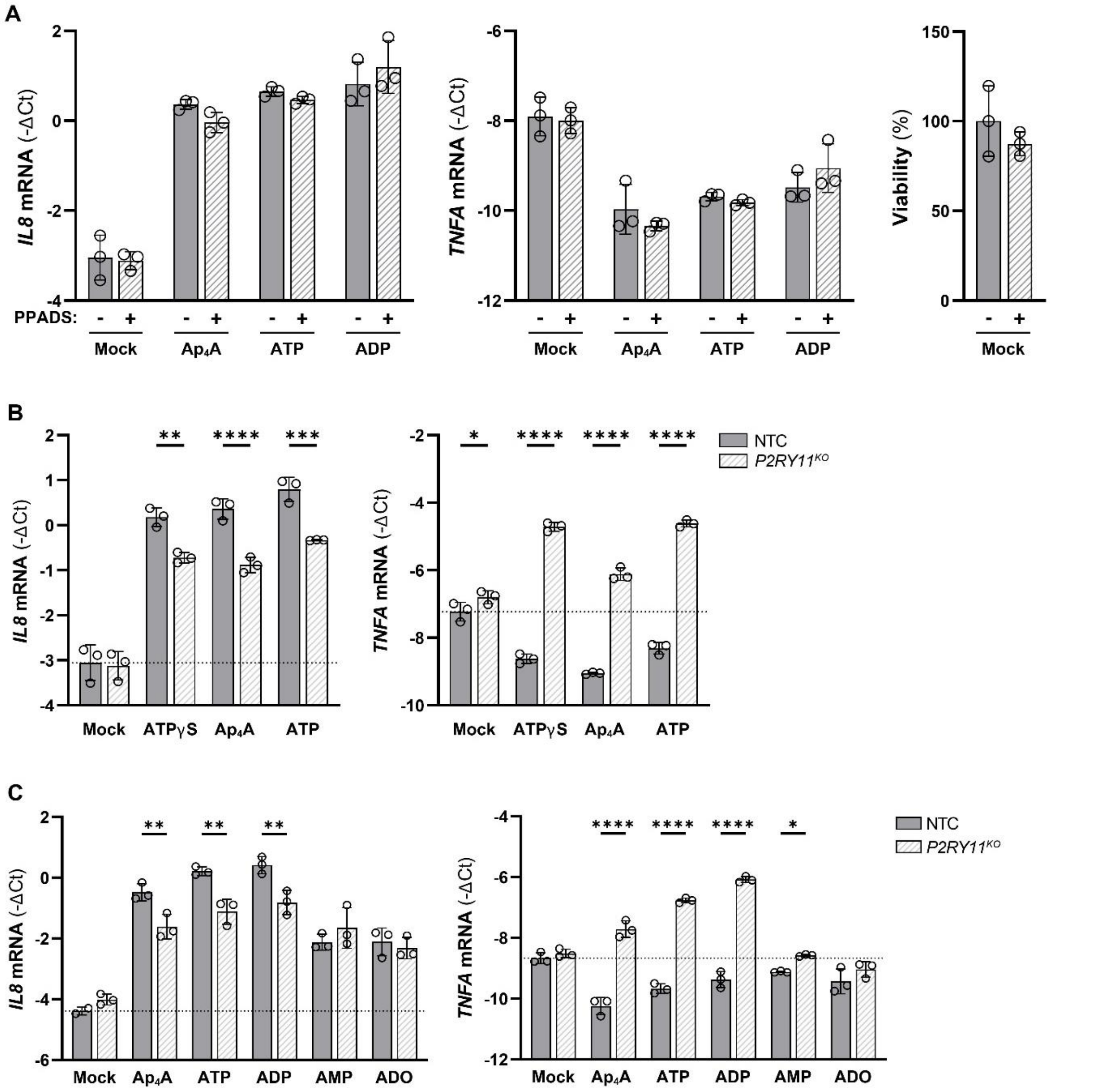

Appendix Figure S6: Functional characterization of P2RY11 knockout cells

(A) Differentiated BLaER1 cell pretreated with 100 µM PPADS for 30 min, then treated with 500 µM Ap_4A, ATP or ADP for 2 h and the *IL8* and *TNFA* levels analyzed by RT-qPCR. The *IL8* and *TNFA* levels were normalized to *GAPDH* and the mean ±SD of three biological replicates shown. ⅛ of untreated differentiated BLaER1 cells were used for the viability measurement using the CellTiter-Glo assay. The viability is expressed as the percentage of the mock average and the mean ±SD of three biological replicates shown.

(B-C) Differentiated BLaER1 NTC and *P2RY* KO cells treated with 500 µM ATPγS, Ap_4A or ATP (B) or Ap_4A, ATP, ADP, AMP or adenosine (ADO) (C) for 2 h and analyzed for *IL8* and *TNFA* levels using RT-qPCR. The *IL8* and *TNFA* levels were normalized to *GAPDH* and the mean ±SD of three biological replicates shown. ****$p < 0.0001$, ***$p < 0.001$, **$p < 0.01$, *$p < 0.05$ (Two-way ANOVA with Šídák's multiple comparison test). The data presented is a representative of two independent experiments.

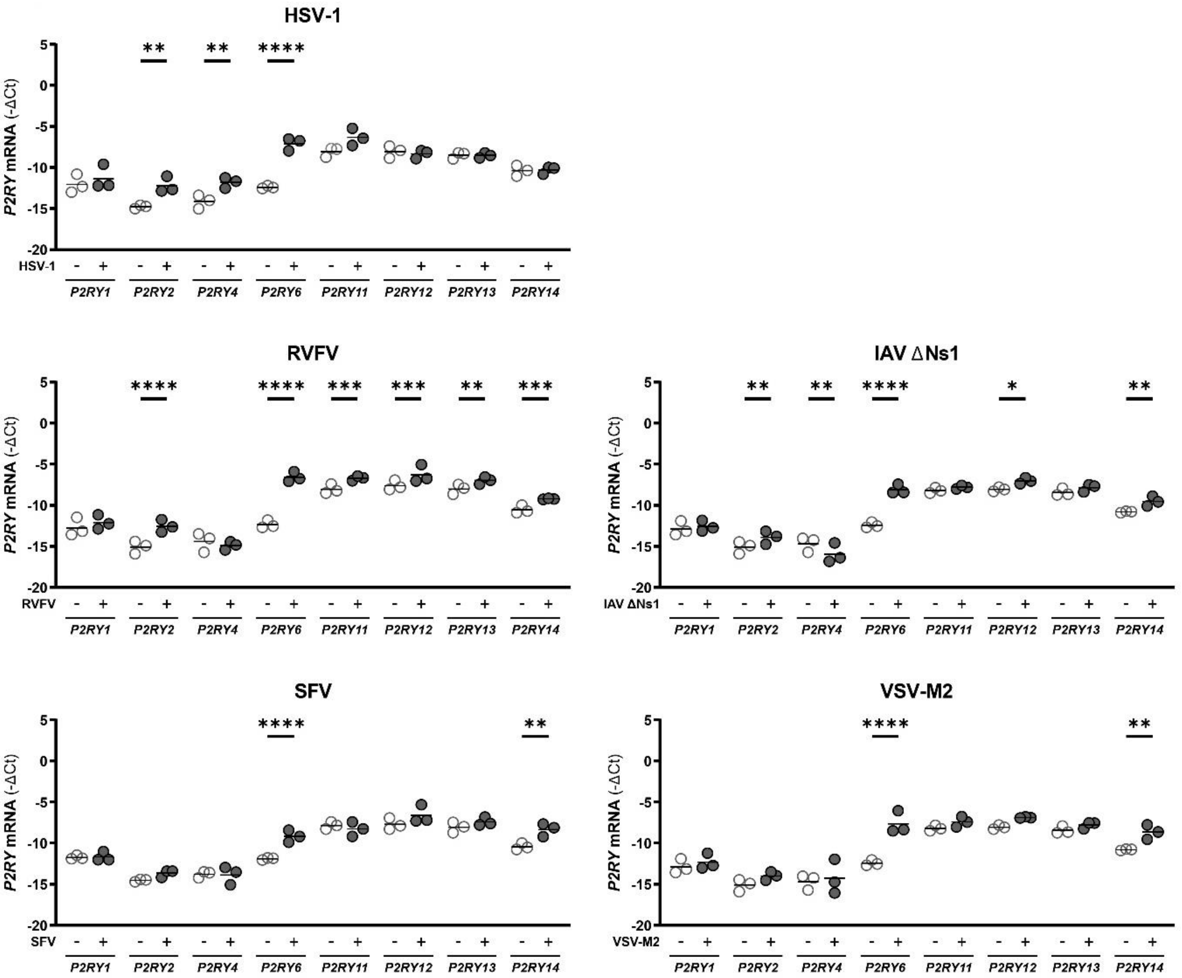

Appendix Figure S7: P2RY expression in virus infected cells

Total RNA from differentiated BLaER1 cells uninfected or infected with HSV-1 (MOI 0.5 for 48 h), RVFV clone 13 (MOI 0.25 for 18 h), IAV S35M ΔNs1 (MOI 0.5 for 16 h), SFV (MOI 0.1 for 18h), VSV-M2 (MOI 0.01 for 18 h) were analyzed for the endogenous *P2RY* levels by RT-qPCR. The *P2RY* levels were normalized to *GAPDH* and the mean ±SD of three biological replicates shown. ****$p < 0.0001$, ***$p < 0.001$, **$p < 0.01$, *$p < 0.05$ (Two-way ANOVA with Šídák's multiple comparison test).

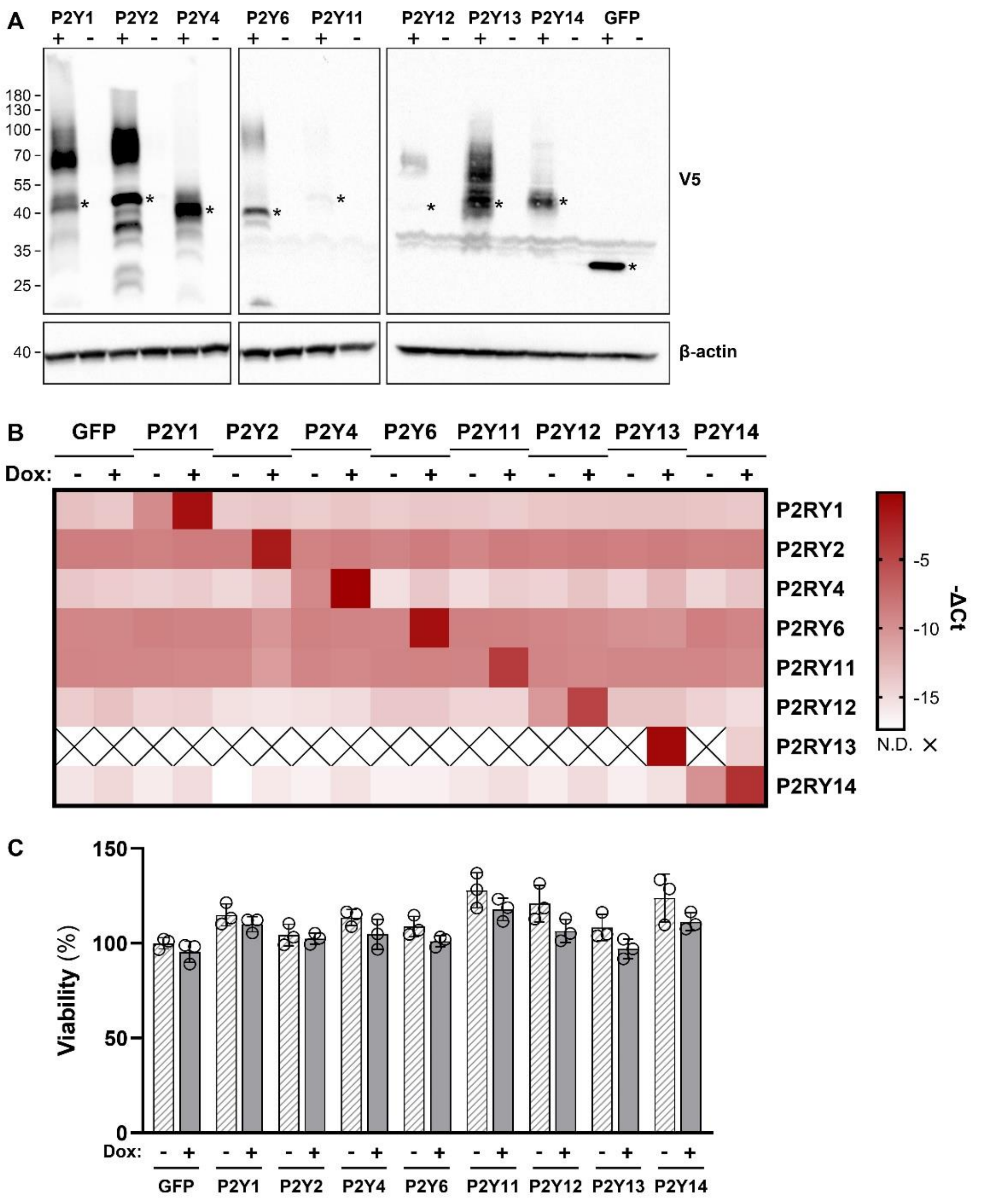

Appendix Figure S8: Inducible P2RY expression in THP-I cells

(A) THP-1 P2YR cells treated with or without 1 µg/mL doxycycline for 24 h and V5-tagged P2YRs and β-actin detected by western blotting.

(B-C) THP-1 P2YR cells differentiated with 100 ng/mL PMA for 48 h and 1 µg/mL doxycycline added on top after the initial 24 h. The *P2RY* levels were analyzed by RT-qPCR and normalized to *GAPDH* (B) and the viability measured using the CellTiter-Glo assay (C). The mean ±SD of three biological replicates is shown.

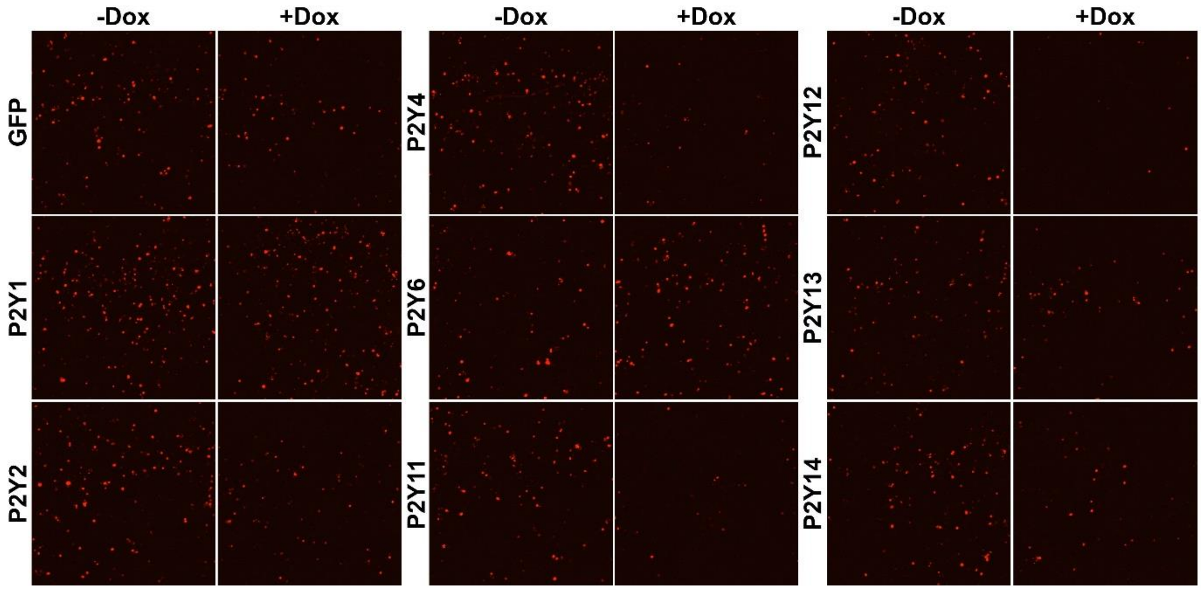

Appendix Figure S9: P2RY expression inhibits SFV growth

Stable THP-1 P2RYs cells differentiated with 100 ng/mL PMA either with or without 1 µg/mL doxycycline for 24 h and then infected with SFV-mCherry (MOI 2). The mCherry signal and cell confluence were tracked for 24 hpi using an IncuCyte S3 live imagining platform. Representative images of the SFV mCherry signal at 15 hpi.
