# Supplementary figures and images for: Systematic P2Y receptor survey identifies P2Y11 as modulator of immune responses and virus replication in macrophages
Source: EMBO J. 2023 Oct 26;42(23):e113279. doi: 10.15252/embj.2022113279 (PMC10690470; doi:10.15252/embj.2022113279)

kDa

180

130

100

70

55

40

35

25

15

10

**ATP**

**LPS**

0 5 10 15 20 30 60 30 min

min

I $\kappa$ B $\alpha$

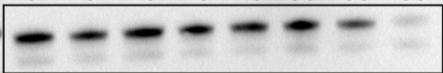

Supplement: Supplementary file 10 — Source Data for Figure 5 [file EMBJ-42-e113279-s007.zip › Figure_5/5B/5B_Western_IkBa.pdf]

kDa

180

130

100

70

55

40

35

25

15

10

**ATP**

**LPS**

0

5

10

15

20

30

60

30

min

$\beta$ -acti

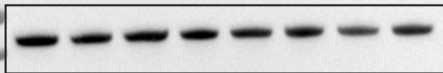

Supplement: Supplementary file 10 — Source Data for Figure 5 [file EMBJ-42-e113279-s007.zip › Figure_5/5B/5B_Western_IkBa_Actin.pdf]

kDa

180

130

100

70

55

40

35

25

15

10

ATP

LPS

0

5

10

15

20

30

60

30

min

p65

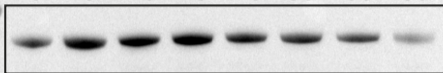

Supplement: Supplementary file 10 — Source Data for Figure 5 [file EMBJ-42-e113279-s007.zip › Figure_5/5B/5B_Western_p65.pdf]

kDa

180

130

100

70

55

40

35

25

15

10

ATP

LPS

0

5

10

15

20

30

60

30

min

 $\beta$ -actin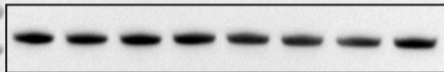

Supplement: Supplementary file 10 — Source Data for Figure 5 [file EMBJ-42-e113279-s007.zip › Figure_5/5B/5B_Western_p65_Actin.pdf]

kDa

ATP

LPS

180

130

100

70

55

40

35

25

15

10

0

5

10

15

20

30

60

30

min

pp65

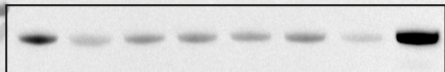

Supplement: Supplementary file 10 — Source Data for Figure 5 [file EMBJ-42-e113279-s007.zip › Figure_5/5B/5B_Western_pp65.pdf]

kDa

180

130

100

70

55

40

35

25

15

**ATP**

**LPS**

0

5

10

15

20

30

60

30

min

ERK1/2

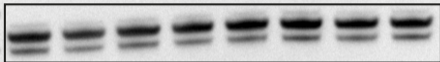

Supplement: Supplementary file 11 — Source Data for Figure 6 [file EMBJ-42-e113279-s008.zip › Figure_6/6D/6D_Western_ERK.pdf]

kDa

180

130

100

70

55

40

35

25

15

**ATP**

**LPS**

0

5

10

15

20

30

60

30

min

$\beta$ -actin

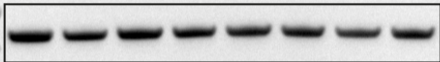

Supplement: Supplementary file 11 — Source Data for Figure 6 [file EMBJ-42-e113279-s008.zip › Figure_6/6D/6D_Western_ERK_Actin.pdf]

kDa

180

130

100

70

55

40

35

25

15

10

ATP

LPS

0

5

10

15

20

30

60

30

min

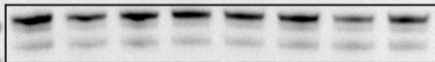

SAPK/JNK

Supplement: Supplementary file 11 — Source Data for Figure 6 [file EMBJ-42-e113279-s008.zip › Figure_6/6D/6D_Western_JNK.pdf]

kDa

180

130

100

70

55

40

35

25

15

10

**ATP**

**LPS**

0

5

10

15

20

30

60

30

min

$\beta$ -actin

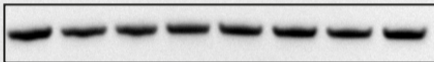

Supplement: Supplementary file 11 — Source Data for Figure 6 [file EMBJ-42-e113279-s008.zip › Figure_6/6D/6D_Western_JNK_Actin.pdf]

kDa

180

130

100

70

55

40

35

25

15

10

**ATP**

**LPS**

0

5

10

15

20

30

60

30

min

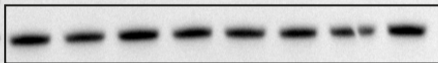

p38

Supplement: Supplementary file 11 — Source Data for Figure 6 [file EMBJ-42-e113279-s008.zip › Figure_6/6D/6D_Western_p38.pdf]

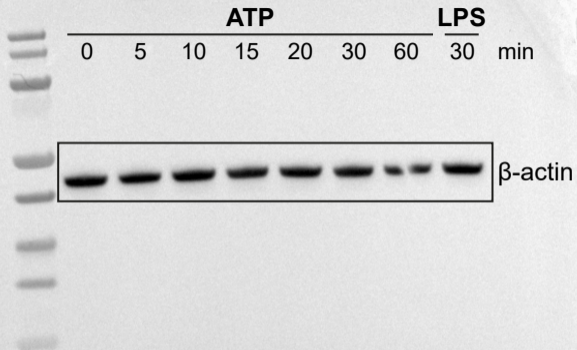

Supplement: Supplementary file 11 — Source Data for Figure 6 [file EMBJ-42-e113279-s008.zip › Figure_6/6D/6D_Western_p38_Actin.pdf]

kDa

180

130

100

70

55

40

35

25

15

**ATP**

**LPS**

0

5

10

15

20

30

60

30

min

pERK1/2

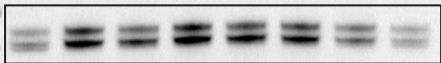

Supplement: Supplementary file 11 — Source Data for Figure 6 [file EMBJ-42-e113279-s008.zip › Figure_6/6D/6D_Western_pERK.pdf]

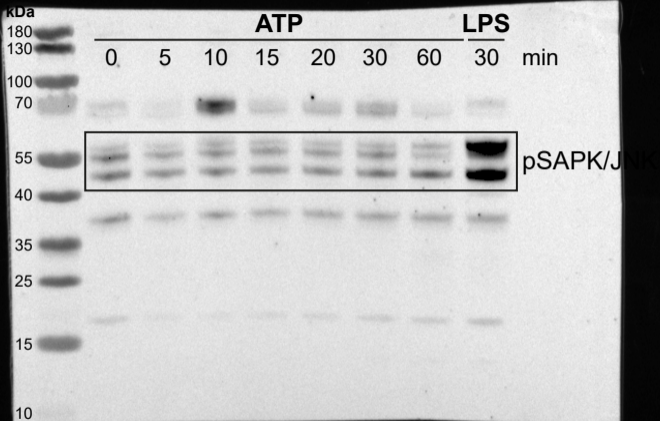

Supplement: Supplementary file 11 — Source Data for Figure 6 [file EMBJ-42-e113279-s008.zip › Figure_6/6D/6D_Western_pJNK.pdf]

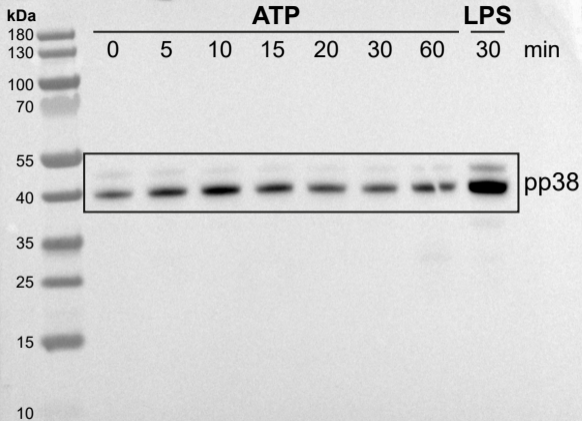

Supplement: Supplementary file 11 — Source Data for Figure 6 [file EMBJ-42-e113279-s008.zip › Figure_6/6D/6D_Western_pp38.pdf]

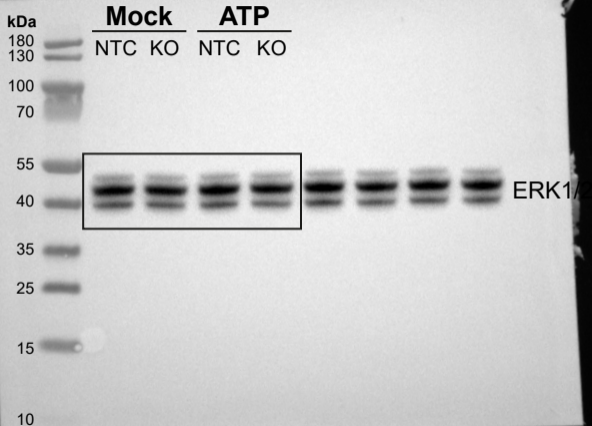

Supplement: Supplementary file 12 — Source Data for Figure 7 [file EMBJ-42-e113279-s011.zip › Figure_7/7D/7D_Western_ERK.pdf]

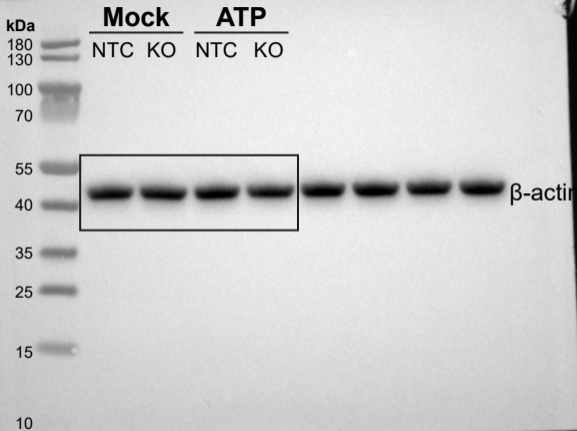

Supplement: Supplementary file 12 — Source Data for Figure 7 [file EMBJ-42-e113279-s011.zip › Figure_7/7D/7D_Western_ERK_Actin.pdf]

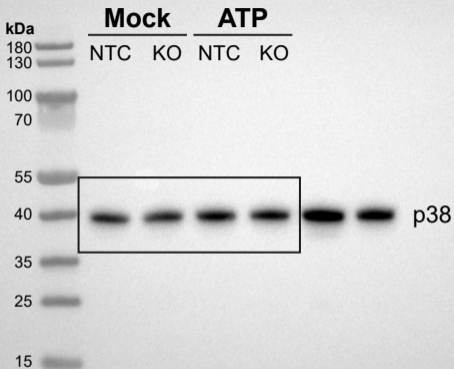

Supplement: Supplementary file 12 — Source Data for Figure 7 [file EMBJ-42-e113279-s011.zip › Figure_7/7D/7D_Western_p38.pdf]

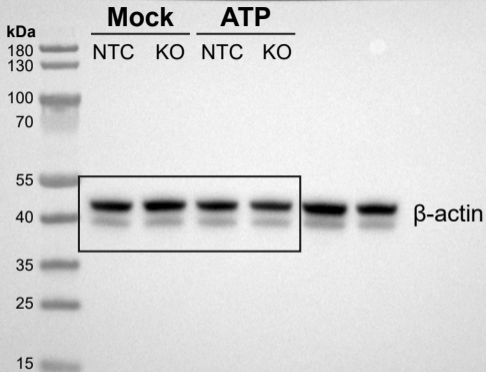

Supplement: Supplementary file 12 — Source Data for Figure 7 [file EMBJ-42-e113279-s011.zip › Figure_7/7D/7D_Western_p38_Actin.pdf]

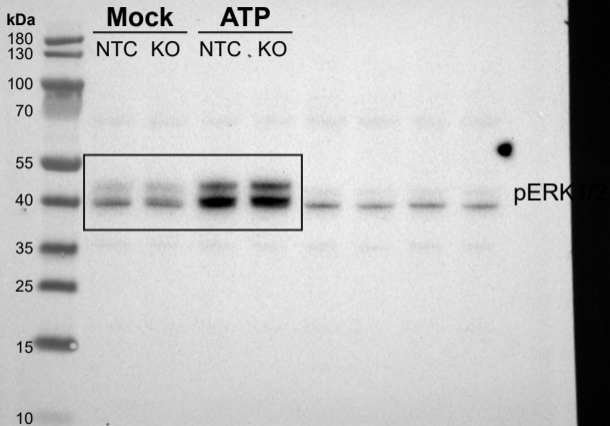

Supplement: Supplementary file 12 — Source Data for Figure 7 [file EMBJ-42-e113279-s011.zip › Figure_7/7D/7D_Western_pERK.pdf]

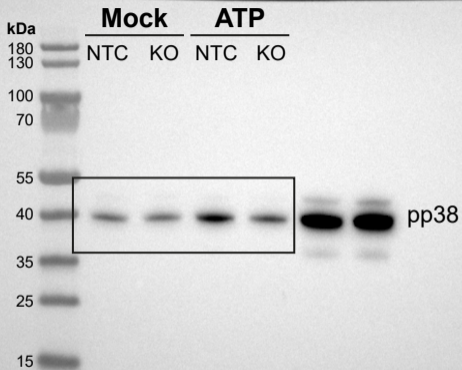

Supplement: Supplementary file 12 — Source Data for Figure 7 [file EMBJ-42-e113279-s011.zip › Figure_7/7D/7D_Western_pp38.pdf]
