# Supplementary material for: Systematic P2Y receptor survey identifies P2Y11 as modulator of immune responses and virus replication in macrophages
Source: EMBO J. 2023 Oct 26;42(23):e113279. doi: 10.15252/embj.2022113279 (PMC10690470; doi:10.15252/embj.2022113279)
Supplement: Supplementary file 13 [file EMBJ-42-e113279-s001.pdf]

THE EMBO JOURNAL

Systematic P2Y receptor survey identifies P2Y11 as modulator of immune responses and virus replication in macrophages

Line Andersen, Yiqi Huang, Christian Urban, Lila Oubraham, Elena Winheim, Che Stafford, Dennis Nagl, Fionan O'Duill, Thomas Ebert, Thomas Engleitner, Søren Paludan, Anne Krug, Roland Rad, Veit Hornung, and Andreas Pichlmair
DOI: 10.15252/embj.2022113279

Corresponding author(s): Andreas Pichlmair (andreas.pichlmair@tum.de) , Line Andersen (line.andersen@tum.de)

Review Timeline:

Submission Date:	14th Dec 22
Editorial Decision:	9th Mar 23
Revision Received:	14th Jul 23
Editorial Decision:	7th Sep 23
Revision Received:	20th Sep 23
Accepted:	29th Sep 23

Editor: Karin Dumstrei

Transaction Report:

(Note: With the exception of the correction of typographical or spelling errors that could be a source of ambiguity, letters and reports are not edited. Depending on transfer agreements, referee reports obtained elsewhere may or may not be included in this compilation. Referee reports are anonymous unless the Referee chooses to sign their reports.)

1st Editorial Decision 9th Mar 2023

Dear Andreas,

Thank you for sending me your response to the referee comments. I have now had a chance to look at it and I appreciate the response. I would therefore like to invite you to submit a revised version carrying out the experiments as outlined in your response.

I do think it will be important to address ref #3 concern #5 (mechanism of action of P2Y11). We don't need the full mechanism but some additional insight is needed. In your response you mention that you can add some omics dataset, but it is not so clear what exactly that is.

When preparing your letter of response to the referees' comments, please bear in mind that this will form part of the Review Process File, and will therefore be available online to the community. For more details on our Transparent Editorial Process, please visit our website: https://www.embopress.org/page/journal/14602075/authorguide#transparentprocess

Let me know if we need to discuss anything further.

best Karin

Karin Dumstrei, PhD
Senior Editor
The EMBO Journal

Instructions for preparing your revised manuscript:

Please make sure you upload a letter of response to the referees' comments together with the revised manuscript.

Please also check that the title and abstract of the manuscript are brief, yet explicit, even to non-specialists.

When assembling figures, please refer to our figure preparation guideline in order to ensure proper formatting and readability in print as well as on screen:
https://bit.ly/EMBOPressFigurePreparationGuideline
See also guidelines for figure legends: https://www.embopress.org/page/journal/14602075/authorguide#figureformat

At EMBO Press we ask authors to provide source data for the main manuscript figures. Our source data coordinator will contact you to discuss which figure panels we would need source data for and will also provide you with helpful tips on how to upload and organize the files.

IMPORTANT: When you send the revision we will require
- a point-by-point response to the referees' comments, with a detailed description of the changes made (as a word file).
- a word file of the manuscript text.
- individual production quality figure files (one file per figure)
- a complete author checklist, which you can download from our author guidelines (https://www.embopress.org/page/journal/14602075/authorguide).
- Expanded View files (replacing Supplementary Information)
Please see out instructions to authors
https://www.embopress.org/page/journal/14602075/authorguide#expandedview

Please remember: Digital image enhancement is acceptable practice, as long as it accurately represents the original data and conforms to community standards. If a figure has been subjected to significant electronic manipulation, this must be noted in the figure legend or in the 'Materials and Methods' section. The editors reserve the right to request original versions of figures and the original images that were used to assemble the figure.

Further information is available in our Guide For Authors: https://www.embopress.org/page/journal/14602075/authorguide

We realize that it is difficult to revise to a specific deadline. In the interest of protecting the conceptual advance provided by the work, we recommend a revision within 3 months (7th Jun 2023). Please discuss the revision progress ahead of this time with the editor if you require more time to complete the revisions.

As a matter of policy, competing manuscripts published during this period will not negatively impact on our assessment of the

conceptual advance presented by your study. However, we request that you contact the editor as soon as possible upon publication of any related work, to discuss how to proceed.

Use the link below to submit your revision:

https://emboj.msubmit.net/cgi-bin/main.plex

Referee #1:

The manuscript entitled "A P2Y Receptor survey identifies P2Y11 as an immunomodulatory and antiviral protein in macrophages" by Andersen et al. reports novel findings about the role of P2Y receptors (P2YRs) in inflammatory and antiviral response in in-vitro models of macrophages. In particular, the authors investigate the P2YRs-mediated response to extracellular adenine- and uridine-based nucleotides, identifying two functional subsets of P2YRs: the inflammatory subset that regulates cytokines induction in an AP-1 dependent manner and another one with direct antiviral potential. Only P2Y11 receptor belongs to both subgroups, being able to induce inflammatory response and being involved in antiviral restriction in macrophages.
This is a well written and documented work with a good relevance to the field.
The manuscript would benefit from the following:
- As well-known and mentioned in the introduction, P2X receptors (as P2X7) when activated by ATP are involved in immune-related inflammatory response and in the released of several cytokines and chemokines. Have authors considered this in their models? What is the contribution of P2X receptors into the inflammatory response after ATP treatment reported by the authors? It would help to further dissect the specific inflammatory response mediated by the P2YRs.
- Transcriptome analyses after stimulation with different adenine- and uridine-based nucleotides are reported only for the BLaER1 cells. I suggest to add at least another model (as THP1 that are already used by the authors in the experiments reported in Figure 1).
- Authors reported an increased expression of several cytokines after treatment with different adenine- and uridine-based nucleotides but data are only validated at mRNA levels, except for IL8 that is evaluated at protein level in PBMCs isolated from six donors. It should be interesting to evaluate, at least after adenine-derived nucleotides treatment, if there is an increase in release of IL1B, IL6 or TNFa both in BLaER1 cells and in PBMCs derived from donors.

Referee #2:

The manuscript written by Andersen, et al. described a P2Y receptor survey identifies P2Y11 as an immunomodulatory and antiviral protein in macrophages. The authors have systematically investigated macrophage responses to extracellular nucleotides focusing on the P2Y receptor family. The data showed that adenine- and uridine-based nucleotides induce a specific, immediate and transient cytokine response through the MAPK signaling pathway regulation of AP-1 transcriptional activation. Especially the study found that P2Y11 was associated with both inflammatory and antiviral responses. In general, the manuscript is well written and the data is encouraging and important, but there are several concerns need to be addressed.
1. For the transcriptome analysis in Fig.3A, 5A, have the authors analyzed the negative control (0h)? if it has been done, the data of 0h should be shown in the figures.
2. In the study, ATP could affect the MAPK signaling pathway, but whether P2Y11 also affect the MAPK signaling pathway has not been defined. P2Y11 KO cells should be used to analyze the signaling pathway.
3. In Fig.5, ATP could activate ERK and P38 signaling pathway, but the study has not verified that if the signaling pathway was associated with the pro-inflammatory cytokine expression. The inhibitors of MAPK signaling pathway could be used to define it.
4. In the study, the data have shown that P2Y11 affected the expression of the pro-inflammatory cytokines such as IL-8, TNF-α, and P2Y11 also had the antiviral activity, whether the expression of the pro-inflammatory cytokines was associated with the antiviral activity?
5. Some grammar and spelling should be addressed.
For example: Line 259, "doxycycline treated cells" should be "doxycycline-treated cells"
Line 263, "in a nucleotide-treatment dependent manner" should be "in a nucleotide treatment-dependent manner"
Line 317, "in GFP expressing cells" should be "in GFP-expressing cells"
Line 369, "on mRNA level"should be "at mRNA level"

Referee #3:

Andersen and others systematically investigated the function and mechanism of P2RY family members in inflammation and antiviral immune responses. They demonstrated that P2RYs are highly expressed in immune cells and some of them could be

induced by IFN-α suggested the potential role of P2RYs in viral infection. Furthermore, they also showed the function of P2YR ligands in regulation of cytokine production and downstream signaling pathway. Most importantly, they firstly reported the predominant function of P2RY11 in restricting viral infection which generated some intriguing observations and added insights to the field. Although the topic is interesting and a lot of work has been done to explore the role of P2RYs, it still has some major concerns need to be addressed.

1. In order to explore the potential role of P2RYs in viral infection, the authors treated the cells with IFN and found that only the expression of P2RY6 and P2RY14 are significantly increased. Actually, that will be interesting to check the expression change in real viral infection. So that I will appreciate if the authors could demonstrate the expression of P2RYs in clinical viral infection which will increase the clinical relevance of them significantly. For example, you can check the clinical data base to see the expression of them in COVID-19 patients. Or you can infect the cells with SFV to mimic the process of viral infection.

2. In this study, the authors treated the cells with Ap4A, ATP, ADP, UTP and UDP to investigate the function and signaling of P2RYs. But as I know the stability of extracellular nucleotides is very low which could be hydrolyzed to adenosine by CD39/CD73. How to exclude the role of adenosine in this study? Have you tried ATP-γ-s to replace ATP which is hydrolyzed very slowly by phosphatases and most ATPases.

3. In Figure 6A, the expression of IL8 was only influenced in P2RY11 knockout cells. But I couldn't see any data about the expression of P2RYs in each knockout cells. So, we need some data to make sure the P2RYs are knockout as expected and the influence on the other P2RYs in these knockout cells should be demonstrated as well. Otherwise, I would like to see the influence of different P2RYs inhibitors on IL8 production.

4. In viral infection assay, only the overexpression cells were used to test the function of P2RYs in SFV infection. So that will be important to do the same assay in P2RYs knockout cells which will strength the conclusion significantly. Meanwhile, only one kind of RNA virus is not enough to demonstrate the role of P2RY11 in viral infection. We don't know whether the influence is only limited to RNA virus without the data on the other virus such as DNA virus.

5. Although the authors showed the function and downstream signaling of P2RYs in many ways, most of them have been reported previously. Such as the function of P2RY13 and the role of AP-1 in P2RYs signaling. Whereas, the role of P2RY11 in restricting viral infection hasn't been reported before so this could be the most attracting to the readers. So, we need more data on how the viral infection is influenced by P2RY11. The transcriptomics analysis of P2RY11 overexpressed or knockout cells in viral infection will be helpful to explore the unique mechanism of P2RY11.

6. Generally, the figure should be numbered according to their appearance order. But the Fig4A is appeared before Fig3B and Fig3D. And this problem should be fixed in the revised manuscript.

Referee #1

The manuscript entitled "A P2Y Receptor survey identifies P2Y11 as an immunomodulatory and antiviral protein in macrophages" by Andersen et al. reports novel findings about the role of P2Y receptors (P2YRs) in inflammatory and antiviral response in in-vitro models of macrophages. In particular, the authors investigate the P2YRs-mediated response to extracellular adenine- and uridine-based nucleotides, identifying two functional subsets of P2YRs: the inflammatory subset that regulates cytokines induction in an AP-1 dependent manner and another one with direct antiviral potential. Only P2Y11 receptor belongs to both subgroups, being able to induce inflammatory response and being involved in antiviral restriction in macrophages. This is a well written and documented work with a good relevance to the field. The manuscript would benefit from the following:

We want to thank reviewer 1 for the positive evaluation and the encouraging feedback.

- As well-known and mentioned in the introduction, P2X receptors (as P2X7) when activated by ATP are involved in immune-related inflammatory response and in the released of several cytokines and chemokines. Have authors considered this in their models? What is the contribution of P2X receptors into the inflammatory response after ATP treatment reported by the authors? It would help to further dissect the specific inflammatory response mediated by the P2YRs.

Reviewer 1 is right by mentioning that activation of a number of P2X receptors, especially $P2X_7$, with their ligand ATP previously have been associated with immune responses. This means that when stimulating macrophages with nucleotides such as ATP there is the possibility of also activating additional nucleotide sensitive receptors such as members of the P2XR family. To address the possible P2XR contribution to the observed transcriptional response, we used the pan P2R inhibitor PPADS. PPADS is known to inhibit several P2Rs of the P2XR and P2YR families. One of the exceptions is $P2Y_{11}$, which upon the discovery was proven insensitive to PPADS (Communi, Robaye, and Boeynaems 1999). Even at a relatively high concentration of PPADS (100 µM) there is no effect on either the upregulation of *IL8* or the downregulation of *TNFA* mRNA in response to adenine-derived nucleotides (Fig. S6a). Moreover, the pretreatment with a specific $P2X_7$ inhibitor A438079 at a concentration that has previously been shown to inhibit inflammasome activation (10 µM) in BLaER1 cells (Gaidt et al. 2016), did not affect the transcriptional responses induced by adenine-derived nucleotides either (Fig. S3b).
These data are in line with a specific and prominent role of $P2Y_{11}$ in the induction of cytokines in response to ATP treatment. A profound analysis employing genetic targeting of diverse P2XRs would require substantial amount of work and a manuscript comparable to the one we submitted here. However, our experiments using P2XR Inhibitors indicate that the contribution of P2XRs to cytokine expression probably is minimal.

- Transcriptome analyses after stimulation with different adenine- and uridine-based nucleotides are reported only for the BLaER1 cells. I suggest to add at least another model (as THP1 that are already used by the authors in the experiments reported in Figure 1).

We agree that testing additional cellular models would substantiate the depth of the discoveries in our manuscript. We had already added data on Ap_4A-treated primary macrophages, which

recapitulated the response we describe in BLaER1 macrophages (Fig. 3D). In initial experiments, we also stimulated differentiated THP-1 cells and tested them for their response to nucleotides. In principle, THP-1 cells give a similar response to BLaER1 cells (Reviewer Fig. 1A). The adenine-derived nucleotides upregulated *IL8* and *IL1B,* and we could confirm downregulation of *TNFA* in Ap_4A and ADP stimulated THP-1-derived macrophages.
However, the challenge with the THP-1 cells is the considerable variation in responsiveness between independent experiments. We realized that THP-1s change their inflammatory potential upon cultivating for a number of passages and that can be appreciated in the basal cytokine expression in mock-treated THP-1 macrophages from four independent experiments (Reviewer Fig. 1B). For that reason, we opted not to use THP-1 cells for validation experiments. In contrast, the nucleotide-response of BLaER1 cells is highly reproducible and we therefore opted to use this model to characterize nucleotide and P2YR dependent signaling.

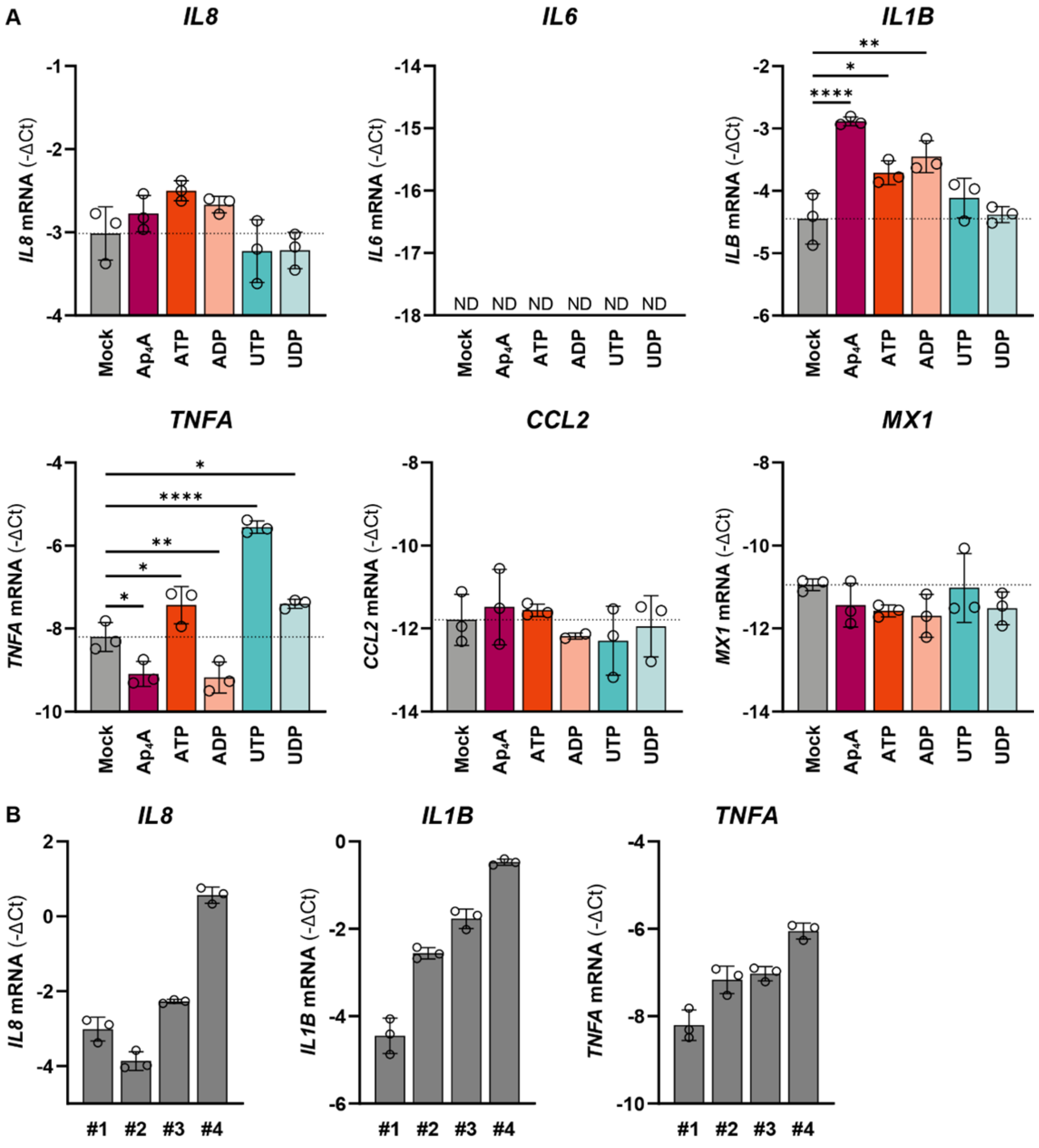

Reviwer Figure 1: THP-1 inflammation. (A) THP-1 cells differentiated with 100 ng/mL PMA for 48 h treated with 500 µM Ap_4A, ATP, ADP, UTP and UDP for 2 h and the *IL8*, *IL6*, *IL1B*, *TNFA*, *CCL2* and *MX1* levels analyzed by RT-qPCR. **(B)** The *IL8*, *IL1B* and *TNFA* levels from 4 independent experiments performed as described in (A) with the numbers indicating increasing passage after thawing. Experiment #1 is the experiment depichted in full in (A). The cytokine levels were normalized to *GAPDH* and the mean ±SD of three biological replicates shown.

- Authors reported an increased expression of several cytokines after treatment with different adenine- and uridine-based nucleotides but data are only validated at mRNA levels, except for IL8 that is evaluated at protein level in PBMCs isolated from six donors. It should be interesting to evaluate, at least after adenine-derived nucleotides treatment, if there is an increase in release of IL1B, IL6 or TNFa both in BLaER1 cells and in PBMCs derived from donors.

This is a very valid point and we thus profiled cytokine secretion in BLaER1 macrophages upon treatment with adenine- and uridine-derived nucleotides using a cytometric bead array (CBA). The results from the CBA are in line with the mRNA profile confirming that the transcriptional regulation also translates into altered cytokine release. The new data is now shown in Fig. 4 of the revised manuscript. We believe that this additional data supports our model on nucleotide stimulated cytokine responses.

Referee #2

The manuscript written by Andersen, et al. described a P2Y receptor survey identifies P2Y11 as an immunomodulatory and antiviral protein in macrophages. The authors have systematically investigated macrophage responses to extracellular nucleotides focusing on the P2Y receptor family. The data showed that adenine- and uridine-based nucleotides induce a specific, immediate and transient cytokine response through the MAPK signaling pathway regulation of AP-1 transcriptional activation. Especially the study found that P2Y11 was associated with both inflammatory and antiviral responses. In general, the manuscript is well written and the data is encouraging and important, but there are several concerns need to be addressed.

We thank reviewer #2 for his thorough review, which further improved the manuscript.

1. For the transcriptome analysis in Fig.3A, 5A, have the authors analyzed the negative control (0h)? if it has been done, the data of 0h should be shown in the figures.

In the transcriptome analysis presented in Fig. 2, 3A, 5A (now 6A in the revised manuscript) and 5B (now 6B in the revised manuscript) a 0 h time point was not included in the experimental setup. The data is presented as transcript expression changes as compared to mock treated cells harvested at the same time after stimulation. Prior to normalization, we did test that mock treated samples did not significantly differ in their gene expression profile in the 1-6 h timespan of the experiment (Fig. 2a). The analysis is described in material and methods and the figure legend.

2. In the study, ATP could affect the MAPK signaling pathway, but whether P2Y11 also affect the

MAPK signaling pathway has not been defined. P2Y11 KO cells should be used to analyze the signaling pathway.

This is an important point, which we have now addressed by analyzing the ERK1/2 and p38 activation by phosphorylation in NTC vs *P2RY11* KO cells in response to ATP using western blotting. We could confirm that *P2RY11* depletion leads to reduced p38 phosphorylation, as compared to stimulation of NTC cells (new Fig. 7D in the revised manuscript). These additional data show that p38 is active downstream of $P2Y_{11}$ and proves that $P2Y_{11}$ is indeed regulating MAPK signaling after engaging adenosine derived nucleotides.

3. In Fig.5, ATP could activate ERK and P38 signaling pathway, but the study has not verified that if the signaling pathway was associated with the pro-inflammatory cytokine expression. The inhibitors of MAPK signaling pathway could be used to define it.

This is a valid point that we tested extensively during revision of this manuscript. To verify the association between the pro-inflammatory cytokine expression, we pretreated differentiated BLaER1 cells with a MEK inhibitor (Binimetinib) and a p38 inhibitor (SB2390063) prior to treatment with ATP. Unfortunately, it was difficult to assess the true effect of the inhibitors due to the effects of the p38 and MEK inhibitors on the basal transcription (0 µM) of *IL8* and *TNFA*, respectively. Due to these oberservations we would prefer not to add these data to the manuscript.

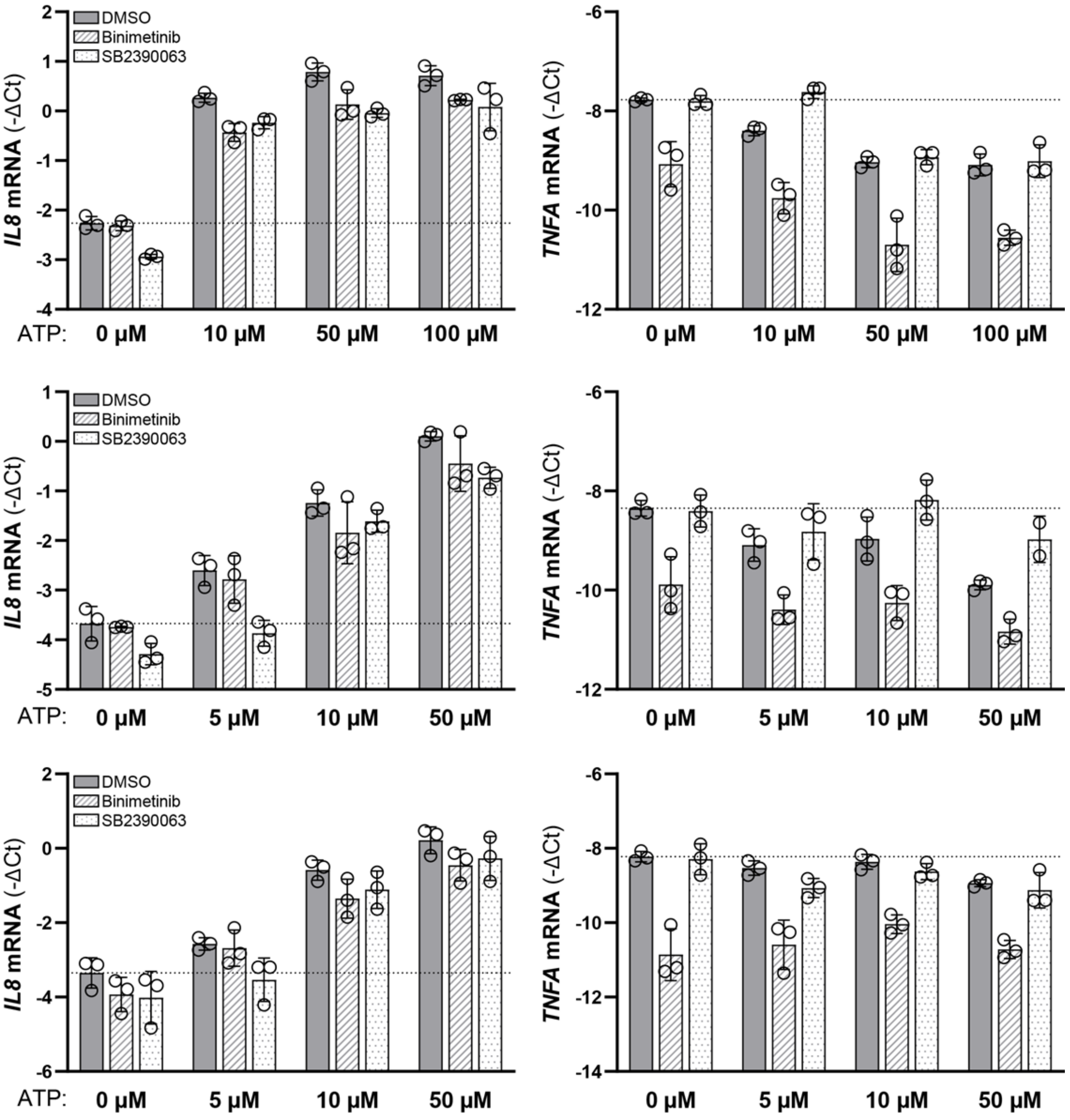

Reviwer Figure 2: MAPK inhibition. Differentiated BLaER1 cells were pretreated with 10 µM Binimetinib or SB2390063 for 4 h prior to treatment with increasing concentration of ATP and the levels of the indicated cytokines analyzed by RT-qPCR. The cytokine levels were normalized to *GAPDH* and the mean ±SD of three biological replicates shown.

4. In the study, the data have shown that P2Y11 affected the expression of the pro-inflammatory cytokines such as IL-8, TNF-α, and P2Y11 also had the antiviral activity, whether the expression of the pro-inflammatory cytokines was associated with the antiviral activity?

In this manuscript, we are using cytokine induction as a readout to test the activity of a $P2Y_{11}$-mediated signaling pathway. We are not claiming that there is a direct link between the ability of $P2Y_{11}$ to induce cytokines and the observed effect on virus replication. We cannot exclude that $P2Y_{11}$ signaling would affect non-cytokine related functions that are required by the virus. However, in the discussion we suggest that the cytokines could promote the overall antiviral immune response. We

feel that additional experiments such as treating cells with recombinant cytokines to induce antiviral immune responses would be redundant to already published literature. We therefore ask reviewer 2 for his/her understanding that we did not test individual cytokines that we identified to be induced by $P2Y_{11}$ activation.

5. Some grammar and spelling should be addressed.
For example: Line 259, "doxycycline treated cells" should be "doxycycline-treated cells" Line 263, "in a nucleotide-treatment dependent manner" should be "in a nucleotide treatment-dependent manner"
Line 317, "in GFP expressing cells" should be "in GFP-expressing cells"
Line 369, "on mRNA level "should be "at mRNA level"

We highly appreciate the feedback from reviewer 2. We corrected all the above changes to the grammar and spelling.

Referee #3

Andersen and others systematically investigated the function and mechanism of P2RY family members in inflammation and antiviral immune responses. They demonstrated that P2RYs are highly expressed in immune cells and some of them could be induced by IFN-α suggested the potential role of P2RYs in viral infection. Furthermore, they also showed the function of P2YR ligands in regulation of cytokine production and downstream signaling pathway. Most importantly, they firstly reported the predominant function of P2RY11 in restricting viral infection which generated some intriguing observations and added insights to the field. Although the topic is interesting and a lot of work has been done to explore the role of P2RYs, it still has some major concerns need to be addressed.

1. In order to explore the potential role of P2RYs in viral infection, the authors treated the cells with IFN and found that only the expression of P2RY6 and P2RY14 are significantly increased. Actually, that will be interesting to check the expression change in real viral infection. So that I will appreciate if the authors could demonstrate the expression of P2RYs in clinical viral infection which will increase the clinical relevance of them significantly. For example, you can check the clinical data base to see the expression of them in COVID-19 patients. Or you can infect the cells with SFV to mimic the process of viral infection.

This is an interesting point that we now address experimentally in the revised manuscript. As reviewer 3 correctly points out, we show that *P2RY6* and *P2RY14* expression is significantly increased in response to type-I interferon treatment (Fig. 1B, S1A). To investigate the expressional regulation in the setting of viral infection, we infected differentiated BLaER1 cells with a panel of viruses of different classes (Fig. S7). Interestingly we find that infection with SFV and VSV-M2 upregulates *P2RY6* and *P2RY14* similar to type-I interferon treatment, while HSV-1, IAV ΔNs1 and RVFV seem to also regulate additional P2YRs. HSV-1 and IAV ΔNs1 additionally regulates *P2RY2* and *P2RY4* expression, whereas RVFV by far has the broadest regulatory capacity with regulation of all P2YRs except *P2RY1* and *P2RY4* (Fig. S7). This nicely reflects the importance of the P2YRs during different virus infections.

2. In this study, the authors treated the cells with Ap4A, ATP, ADP, UTP and UDP to investigate the function and signaling of P2RYs. But as I know the stability of extracellular nucleotides is very low which could be hydrolyzed to adenosine by CD39/CD73. How to exclude the role of adenosine in this study? Have you tried ATP-γ-s to replace ATP which is hydrolyzed very slowly by phosphatases and most ATPases.

This is a very valid comment. Degradation of nucleotides is always of concern in stimulation assays and this point is particularly relevant for adenine-derived nucleotides since adenosine previously has been reported to influence immune responses. We took two different approaches to test whether nucleotide degradation or degradation products contribute to the signaling events observed after stimulation. As suggested by the reviewer, we first tried ATPγS treatment. ATPγS was similar in activity as ATP, which indicates that the observed response is not due to degradation (Fig. S6B). Additionally, we tested the effect of the ATP degradation products AMP and adenosine (ADO) in the context of $P2Y_{11}$-dependent responses. AMP and ADO induce upregulation of *IL8* and slight downregulation of *TNFA*, but to a lesser than the other adenine-derived nucleotides. The effect of AMP and ADO is comparable to the response to Ap_4A, ATP and ADP in the *P2RY11* knockout cells. This is an interesting result since it may indicate that residual activity seen in Ap_4A, ATP and ADP stimulated *P2RY11* knockout cells may be due to alternative pathways that are stimulated by degradation products. However, most importantly, it shows that the effect of AMP and ADO is independent of $P2Y_{11}$ and is therefore not affecting our interpretations (Fig. S6C).

3. In Figure 6A, the expression of IL8 was only influenced in P2RY11 knockout cells. But I couldn't see any data about the expression of P2RYs in each knockout cells. So, we need some data to make sure the P2Rys are knockout as expected and the influence on the other P2Rys in these knockout cells should be demonstrated as well. Otherwise, I would like to see the influence of different P2Rys inhibitors on IL8 production.

We agree that additional controls for the specificity of the knockout cells are important and therefore added additional data showing the depletion of *P2RY1*, *P2RY11*, *P2RY12* and *P2RY13* on mRNA and, importantly, that the depletion of one receptor does not affect mRNA expression of other P2RY receptors. *P2RY2*, *P2RY4*, *P2RY6* and *P2RY14* were too close to the detection limit and definite conclusions therefore difficult to draw (Fig. S5A). On a genomic level, the knockout approach used will create a deletion that will change the size, when analyzed by PCR amplification of the targeted region followed by visualization on an agarose gel. A shift can be seen in the knockout cells lines for all the targeted P2YRs except *P2RY4* (reviewer Figure 3), supporting specific targeting of the P2YRs. Both the mRNA and genomic analysis suggest that the knockout efficiencies are only partial.

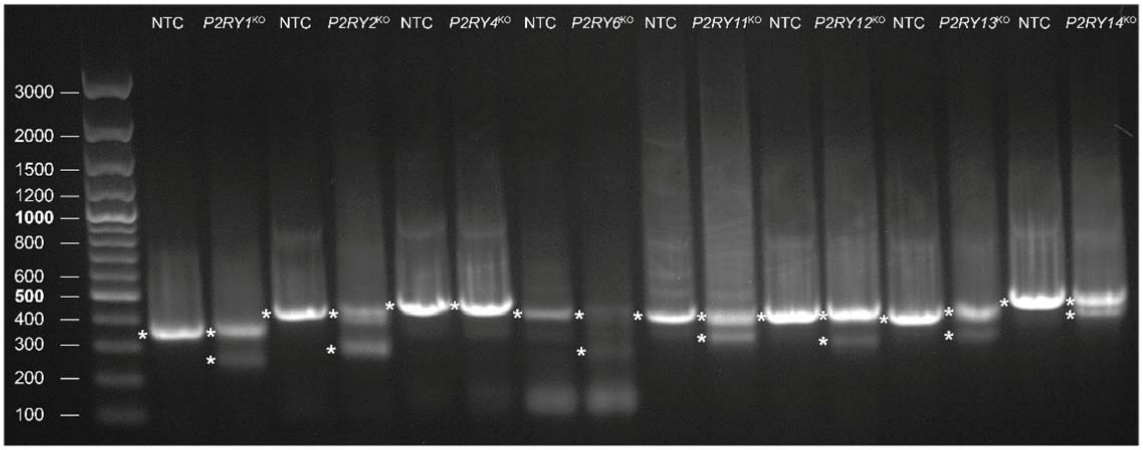

Reviwer Figure 3: Genomic knockout verification. Genomic DNA was isolated from each cell line and the genomic region targeted in the knockouts amplified in the NTC and the corresponding knockout cell line by PCR using specific primers. The PCR products were run on an agarose gel and if the genomic region was sucessfully targted by the knockout approach, a deletion will be created changing the size. The asterixes indicate the expected size with and without a succesful deletion.

4. In viral infection assay, only the overexpression cells were used to test the function of P2RYs in SFV infection. So that will be important to do the same assay in P2RYs knockout cells which will strength the conclusion significantly. Meanwhile, only one kind of RNA virus is not enough to demonstrate the role of P2RY11 in viral infection. We don't know whether the influence is only limited to RNA virus without the data on the other virus such as DNA virus.

We agree with reviewer 3 that validating the response seen in the overexpression cells in the *P2RY* knockout cells would strengthen the conclusion. However, the proposed experiments with infection of *P2RY* knockout cells are complicated since BLaER1 cells are often not very well infected with our tagged viruses, which means that a simple and reliable screening approach using live cell imaging is challenging in this cell type. Using RT-qPCR based readout for different viruses and time-kinetics for all knockouts would be tremendous amount of work. The alternative would be setting up *P2RY* knockout cells in THP-1 background and screening these with the live cell imaging approach, but this alternative approach would be highly time consuming and could not be achieved within the frame of this revision. We therefore ask for the understanding of reviewer 3 that we could not do the requested screen for different viruses in various knockout cells.

Since our manuscript highlights a function of $P2Y_{11}$, we instead chose to elaborate on the effect of $P2Y_{11}$ on virus infection as also requested by reviewer 3. We used the $P2Y_{11}$ inhibitor NF157 to add data on infection with VSV, HSV-1 and RVFV in the context of $P2Y_{11}$ inhibition (Fig. 8B). These data support the antiviral function of $P2Y_{11}$ against SFV. Moreover, $P2Y_{11}$ is also antiviral against the paramyxovirus VSV, although the effect was less prominent. Surprisingly, $P2Y_{11}$ seems to have a pro-viral effect on HSV-1 and RVFV. This is very interesting data and we currently do not know why $P2Y_{11}$ has pro-viral activity for HSV-1 and RVFV. These results are highly interesting and suggest differential requirement of $P2Y_{11}$ in a virus-dependent context and we have added a section were we speculate on possible explanations in the discussion. However, a number of additional experiments would be required to understand the exact anti- and pro-viral mechanisms of $P2Y_{11}$, which we will work on in follow up studies. We ask reviewer 3 for his/her understanding that solving this riddle is too complex for the short revision period.

5. Although the authors showed the function and downstream signaling of P2RYs in many ways, most of them have been reported previously. Such as the function of P2RY13 and the role of AP-1 in P2RYs signaling. Whereas, the role of P2RY11 in restricting viral infection hasn't been reported before so this could be the most attracting to the readers. So, we need more data on how the viral infection is influenced by P2RY11. The transcriptomics analysis of P2RY11 overexpressed or knockout cells in viral infection will be helpful to explore the unique mechanism of P2RY11.

We agree that P2RYs have been linked to signaling and antiviral immunity in some cases. This was also one of the reasons prompting us to study the activity of P2YRs more globally since we felt that there was unmet need for additional comparative information on the activity of P2YRs in the context of macrophages and virus infections. Moreover, the classification of P2YRs in cytokine inducing and non-cytokine inducing receptors is novel and will be highly appreciated by colleagues in the field. We therefore feel that the part of our manuscript describing the P2YR signaling can serve as a highly useful reference to all further P2YR studies.
We agree with reviewer 3 that additional information on the antiviral activity of $P2Y_{11}$ would be interesting. During the revision period, we did transcriptome analysis of virus-infected cells. However, while virus infection induced a multitude of partially very prominent differences, we could not delineate clear P2Y effects in these large datasets. This may be expectable since virus infection is a very dynamic process that induces a very strong perturbation in cells. Getting closer to the molecular mechanism may be difficult in such a complex setting. Since this data is too complex to give the mechanistic insights that we had hoped for we would like to omit these data from the manuscript.
To gain further insights into the activity of $P2Y_{11}$, we performed transcriptome analysis of WT and *P2RY11* knockout cells treated with Ap_4A, ATP and ADP for 2 h. This analysis confirmed that the majority of proteins that we identified to be differentially regulated are due to $P2Y_{11}$ activation. Many of these genes and associated pathways have previously been linked to antiviral immunity and it is therefore likely that the activity of $P2Y_{11}$ can be explained through these activities. We discuss this in the manuscript and hope that reviewer 3 agrees that these additional data is very informative to gain mechanistic insights in the $P2Y_{11}$ activity.

6. Generally, the figure should be numbered according to their appearance order. But the Fig4A is appeared before Fig3B and Fig3D. And this problem should be fixed in the revised manuscript.

We thank reviewer 3 for very carefully reviewing our manuscript. The respective reference to this figure was corrected.

Communi, D., B. Robaye, and J. M. Boeynaems. 1999. 'Pharmacological characterization of the human P2Y11 receptor', *Br J Pharmacol*, 128: 1199-206.
Gaidt, M. M., T. S. Ebert, D. Chauhan, T. Schmidt, J. L. Schmid-Burgk, F. Rapino, A. A. Robertson, M. A. Cooper, T. Graf, and V. Hornung. 2016. 'Human Monocytes Engage an Alternative Inflammasome Pathway', *Immunity*, 44: 833-46.

1st Revision - Editorial Decision 7th Sep 2023

Dear Andreas,

Thank you for submitting your revised manuscript to The EMBO Journal. Your study has now been seen by the three referees. As you can see below, the referees appreciate the introduced changes and support publication here.

I am therefore very pleased to accept the manuscript for publication here. Before sending you, the formal accept letter there are just a few editorial points to sort out:

- We are missing 3-5 keywords

- Reference format: Please use et al after 10 author names

- Tables S1-S6 should be renamed to Dataset EV1-EV6; Table S6 is missing a legend and a title. Please also correct callouts in the MS.

- The supplemental files including their figure legends need to go into an appendix with a ToC. The nomenclature in the file and callouts in the MS should be Appendix Figure S1, etc.

- Please make sure that there is a reference to the uploaded source data for 7E, 7F, 7G, 7H in the MS.

- Please provide the synopsis image in jpeg, TIFF or png format and sized 550 pixels wide x 200-400 pixels high

- We also need a synopsis text with a summary statement plus 3-5 bullet points describing the key findings of the MS

- References should go right Disclosure and competing interest statement

- Our publisher has also done their pre-publication check on your manuscript. When you log into the manuscript submission system you will see the file "Data Edited Manuscript file". They have only some stylistic comments regarding the figure legends. Please incorporate these into the final manuscript.

- Make sure that the deposited datasets are publicly available.

- Take a look at Figure S9: The panels in P2Y1 in -DOX/+DOX looks very similar. The same goes for the panels in P2Y2 +Dox and P2Y4 +DOX.

That should be all!

Best Karin

Karin Dumstrei, PhD
Senior Editor
The EMBO Journal

Instructions for preparing your revised manuscript:

Guide For Authors: https://www.embopress.org/page/journal/14602075/authorguide

Use the link below to submit your revision:

https://emboj.msubmit.net/cgi-bin/main.plex

Referee #1:

All concerns in the first revision have been addressed in the author's revised manuscript.

Referee #2:

The authors have addressed most of the concerns and the manuscript can be accepted.

Referee #3:

The authors have fulfilled all my concerns about the manuscript.

Point by Point reply for EMBOJ-2022-113279R

Dear Karin,

Apologies that we missed to add the point by point reply, which I hereby provide:

- We are missing 3-5 keywords

We now added Keywords to the MS file

- Reference format: Please use et al after 10 author names

References have been amended accordingly

- Tables S1-S6 should be renamed to Dataset EV1-EV6; Table S6 is missing a legend and a title. Please also correct callouts in the MS.

We re-named the datasets and added a legend to Table S6 – we also change the callouts in the MS file

- The supplemental files including their figure legends need to go into an appendix with a ToC. The nomenclature in the file and callouts in the MS should be Appendix Figure S1, etc.

We now provided the Appendix file with Table of contents and renamed the callouts in the manuscript text (Appendix Fig. NN)

- Please make sure that there is a reference to the uploaded source data for 7E, 7F, 7G, 7H in the MS.

The data is referenced in the manuscript. Source data is part of the ENA database identifier PRJEB60753, as specified in the Data availability section

- Please provide the synopsis image in jpeg, TIFF or png format and sized 550 pixels wide x 200-400 pixels high

We uploaded a synopsis figure with the correct dimensions

- We also need a synopsis text with a summary statement plus 3-5 bullet points describing the key findings of the MS

We provided a synopsis text and the requested bullet points

- References should go right Disclosure and competing interest statement

The order of the sections has been updated accordingly

- Our publisher has also done their pre-publication check on your manuscript. When you log into the manuscript submission system you will see the file "Data Edited Manuscript file". They have only some stylistic comments regarding the figure legends. Please incorporate these into the final manuscript.

We implemented the required changes

- Make sure that the deposited datasets are publicly available.

All datasets are now publically available

- Take a look at Figure S9: The panels in P2Y1 in -DOX/+DOX looks very similar. The same goes for the panels in P2Y2 +Dox and P2Y4 +DOX.

Attached to this email I send the revised Appendix file called (Appendix_revise.docx) which contains the correct images. This is the version that should have been uploaded.

2nd Revision - Editorial Decision 29th Sep 2023

Dear Andreas,

Thank you for submitting your revised manuscript to us. I have now had a chance to look at it and all looks good. I am therefore very pleased to accept the manuscript for publication here.

Congratulations on a nice study!

Best Karin

Karin Dumstrei, PhD
Senior Editor
The EMBO Journal

Please note that it is EMBO Journal policy for the transcript of the editorial process (containing referee reports and your response letter) to be published as an online supplement to each paper. If you do NOT want this, you will need to inform the Editorial Office via email immediately. More information is available here:
https://www.embopress.org/page/journal/14602075/authorguide#transparentprocess

Your manuscript will be processed for publication in the journal by EMBO Press. Manuscripts in the PDF and electronic editions of The EMBO Journal will be copy edited, and you will be provided with page proofs prior to publication. Please note that supplementary information is not included in the proofs.

You will be contacted by Wiley Author Services to complete licensing and payment information. The required 'Page Charges Authorization Form' is available here: https://www.embopress.org/pb-assets/embo-site/tej_apc.pdf - please download and complete the form and return to embopressproduction@wiley.com

EMBO Press participates in many Publish and Read agreements that allow authors to publish Open Access with reduced/no publication charges. Check your eligibility: https://authorservices.wiley.com/author-resources/Journal-Authors/open-access/affiliation-policies-payments/index.html

Should you be planning a Press Release on your article, please get in contact with embojournal@wiley.com as early as possible, in order to coordinate publication and release dates.

If you have any questions, please do not hesitate to call or email the Editorial Office. Thank you for your contribution to The EMBO Journal.

** Click here to be directed to your login page: https://emboj.msubmit.net

EMBO Press Author Checklist

Corresponding Author Name: Andreas Pichlmair
Journal Submitted to: The EMBO Journal
Manuscript Number: EMBOJ-2022-113279R

USEFUL LINKS FOR COMPLETING THIS FORM
The EMBO Journal - Author Guidelines
EMBO Reports - Author Guidelines
Molecular Systems Biology - Author Guidelines
EMBO Molecular Medicine - Author Guidelines

Reporting Checklist for Life Science Articles (updated January
This checklist is adapted from Materials Design Analysis Reporting (MDAR) Checklist for Authors. MDAR establishes a minimum set of requirements in transparent reporting in the life sciences (see Statement of Task: 10.31222/osf.io/9sm4x). Please follow the journal's guidelines in preparing your
Please note that a copy of this checklist will be published alongside your article.

Abridged guidelines for figures
1. Data
The data shown in figures should satisfy the following conditions:
- the data were obtained and processed according to the field's best practice and are presented to reflect the results of the experiments in an accurate and unbiased manner.
- ideally, figure panels should include only measurements that are directly comparable to each other and obtained with the same assay.
- plots include clearly labeled error bars for independent experiments and sample sizes. Unless justified, error bars should not be shown for technical
- if n<5, the individual data points from each experiment should be plotted. Any statistical test employed should be justified.
- Source Data should be included to report the data underlying figures according to the guidelines set out in the authorship guidelines on Data

2. Captions
Each figure caption should contain the following information, for each panel where they are relevant:
- a specification of the experimental system investigated (eg cell line, species name).
- the assay(s) and method(s) used to carry out the reported observations and measurements.
- an explicit mention of the biological and chemical entity(ies) that are being measured.
- an explicit mention of the biological and chemical entity(ies) that are altered/varied/perturbed in a controlled manner.
- the exact sample size (n) for each experimental group/condition, given as a number, not a range;
- a description of the sample collection allowing the reader to understand whether the samples represent technical or biological replicates (including how many animals, litters, cultures, etc.).
- a statement of how many times the experiment shown was independently replicated in the laboratory.
- definitions of statistical methods and measures:
  - common tests, such as t-test (please specify whether paired vs. unpaired), simple $\chi 2$ tests, Wilcoxon and Mann-Whitney tests, can be unambiguously identified by name only, but more complex techniques should be described in the methods section;
  - are tests one-sided or two-sided?
  - are there adjustments for multiple comparisons?
  - exact statistical test results, e.g., P values = x but not P values < x;
  - definition of 'center values' as median or average;
  - definition of error bars as s.d. or s.e.m.

Please complete ALL of the questions below.
Select "Not Applicable" only when the requested information is not relevant for your study.

Materials

Newly Created Materials	Information included in the manuscript?	In which section is the information available? (Reagents and Tools Table, Materials and Methods, Figures, Data Availability Section)
New materials and reagents need to be available; do any restrictions apply?	Not Applicable	

Antibodies	Information included in the manuscript?	In which section is the information available? (Reagents and Tools Table, Materials and Methods, Figures, Data Availability Section)
For **antibodies** provide the following information:<br>- Commercial antibodies: RRID (if possible) or supplier name, catalogue number and or/clone number<br>- Non-commercial: RRID or citation	Yes	Material and Methods

DNA and RNA sequences	Information included in the manuscript?	In which section is the information available? (Reagents and Tools Table, Materials and Methods, Figures, Data Availability Section)
**Short novel DNA or RNA including primers, probes**: provide the sequences.	Yes	Table S6

Cell materials	Information included in the manuscript?	In which section is the information available? (Reagents and Tools Table, Materials and Methods, Figures, Data Availability Section)
**Cell lines:** Provide species information, strain. Provide accession number in repository **OR** supplier name, catalog number, clone number, and/**OR** RRID.	Yes	Materials and Methods
**Primary cultures:** Provide species, strain, sex of origin, genetic modification status.	Not Applicable	
Report if the cell lines were recently **authenticated** (e.g., by STR profiling) and tested for mycoplasma contamination.	Yes	All cell lines were tested for mycoplasma

Experimental animals	Information included in the manuscript?	In which section is the information available? (Reagents and Tools Table, Materials and Methods, Figures, Data Availability Section)
**Laboratory animals or Model organisms:** Provide species, strain, sex, age, genetic modification status. Provide accession number in repository **OR** supplier name, catalog number, clone number, **OR** RRID.	Not Applicable	
**Animal observed in or captured from the field:** Provide species, sex, and age where possible.	Not Applicable	
Please detail **housing and husbandry conditions**.	Not Applicable	

Plants and microbes	Information included in the manuscript?	In which section is the information available? (Reagents and Tools Table, Materials and Methods, Figures, Data Availability Section)
**Plants:** provide species and strain, ecotype and cultivar where relevant, unique accession number if available, and source (including location for collected wild specimens).	Not Applicable	
**Microbes:** provide species and strain, unique accession number if available, and source.	Yes	Materials and Methods

Human research participants	Information included in the manuscript?	In which section is the information available? (Reagents and Tools Table, Materials and Methods, Figures, Data Availability Section)
If collected and within the bounds of privacy constraints report on age, sex and gender or ethnicity for all study participants.	Not Applicable	

Core facilities	Information included in the manuscript?	In which section is the information available? (Reagents and Tools Table, Materials and Methods, Figures, Data Availability Section)
If your work benefited from core facilities, was their service mentioned in the acknowledgments section?	Yes	Acknowledgements

Design

Study protocol	Information included in the manuscript?	In which section is the information available? (Reagents and Tools Table, Materials and Methods, Figures, Data Availability Section)
If study protocol has been **pre-registered, provide DOI in the manuscript**. For clinical trials, provide the trial registration number **OR** cite DOI.	Not Applicable	
Report the **clinical trial registration number** (at ClinicalTrials.gov or equivalent), where applicable.	Not Applicable	

Laboratory protocol	Information included in the manuscript?	In which section is the information available? (Reagents and Tools Table, Materials and Methods, Figures, Data Availability Section)
Provide DOI OR other citation details if **external detailed step-by-step protocols** are available.	Not Applicable	

Experimental study design and statistics	Information included in the manuscript?	In which section is the information available? (Reagents and Tools Table, Materials and Methods, Figures, Data Availability Section)
Include a statement about **sample size** estimate even if no statistical methods were used.	Yes	Figure lenged
Were any steps taken to minimize the effects of subjective bias when allocating animals/samples to treatment (e.g. **randomization procedure**)? If yes, have they been described?	Not Applicable	
Include a statement about **blinding** even if no blinding was done.	Not Applicable	
Describe **inclusion/exclusion criteria** if samples or animals were excluded from the analysis. Were the criteria pre-established?<br><br>If sample or data points were omitted from analysis, report if this was due to attrition or intentional exclusion and provide justification.	Yes	Inclusion data described in the source data. Data were only excluded based on technical reasons: quality control etc.
For every figure, are **statistical tests** justified as appropriate? Do the data meet the assumptions of the tests (e.g., normal distribution)? Describe any methods used to assess it. Is there an estimate of variation within each group of data? Is the variance similar between the groups that are being statistically compared?	Yes	

Sample definition and in-laboratory replication	Information included in the manuscript?	In which section is the information available? (Reagents and Tools Table, Materials and Methods, Figures, Data Availability Section)
In the figure legends: state number of times the experiment was **replicated** in laboratory.	Yes	Figure legend
In the figure legends: define whether data describe **technical or biological replicates**.	Yes	Figure legend

Ethics

Ethics	Information included in the manuscript?	In which section is the information available? (Reagents and Tools Table, Materials and Methods, Figures, Data Availability Section)
Studies involving **human participants**: State details of **authority granting ethics approval** (IRB or equivalent committee(s), provide reference number for approval.	Not Applicable	
Studies involving **human participants**: Include a statement confirming that **informed consent** was obtained from all subjects and that the experiments conformed to the principles set out in the WMA Declaration of Helsinki and the Department of Health and Human Services Belmont Report.	Not Applicable	
Studies involving **human participants:** For publication of **patient photos**, include a statement confirming that consent to publish was obtained.	Not Applicable	
Studies involving experimental **animals**: State details of **authority granting ethics approval** (IRB or equivalent committee(s), provide reference number for approval. Include a statement of compliance with ethical regulations.	Not Applicable	
Studies involving **specimen and field samples:** State if relevant **permits** obtained, provide details of authority approving study; if none were required, explain why.	Yes	Material and Methods

Dual Use Research of Concern (DURC)	Information included in the manuscript?	In which section is the information available? (Reagents and Tools Table, Materials and Methods, Figures, Data Availability Section)
Could your study fall under dual use research restrictions? Please check biosecurity documents and list of **select agents and toxins** (CDC): https://www.selectagents.gov/sat/list.htm	Not Applicable	
If you used a select agent, is the security level of the lab appropriate and reported in the manuscript?	Not Applicable	
If a study is subject to dual use research of concern regulations, is the name of the **authority granting approval and reference number** for the regulatory approval provided in the manuscript?	Not Applicable	

Reporting

The MDAR framework recommends adoption of discipline-specific guidelines, established and endorsed through community initiatives. Journals have their own policy about requiring specific guidelines and recommendations to complement MDAR.

Adherence to community standards	Information included in the manuscript?	In which section is the information available? (Reagents and Tools Table, Materials and Methods, Figures, Data Availability Section)
State if relevant guidelines or checklists (e.g., **ICMJE, MIBBI, ARRIVE, PRISMA**) have been followed or provided.	Not Applicable	
For **tumor marker prognostic studies,** we recommend that you follow the **REMARK** reporting guidelines (see link list at top right). See author guidelines, under 'Reporting Guidelines'. Please confirm you have followed these guidelines.	Not Applicable	
For **phase II and III randomized controlled trials**, please refer to the **CONSORT** flow diagram (see link list at top right) and submit the CONSORT checklist (see link list at top right) with your submission. See author guidelines, under 'Reporting Guidelines'. Please confirm you have submitted this list.	Not Applicable	

Data Availability

Data availability	Information included in the manuscript?	In which section is the information available? (Reagents and Tools Table, Materials and Methods, Figures, Data Availability Section)
Have **primary datasets** been deposited according to the journal's guidelines (see 'Data Deposition' section) and the respective accession numbers provided in the Data Availability Section?	Yes	Data Availability
Were **human clinical and genomic datasets** deposited in a public access-controlled repository in accordance to ethical obligations to the patients and to the applicable consent agreement?	Not Applicable	
Are **computational models** that are central and integral to a study available without restrictions in a machine-readable form? Were the relevant accession numbers or links provided?	Yes	Material and Methods
If publicly available data were reused, provide the respective **data citations in the reference list**.	Not Applicable	
